# Genetic Architecture of Multiphasic Growth Covariation as Revealed by a Nonlinear Mixed Mapping Framework

**DOI:** 10.3389/fpls.2021.711219

**Published:** 2021-10-05

**Authors:** Huiying Gong, Xiao-Yu Zhang, Sheng Zhu, Libo Jiang, Xuli Zhu, Qing Fang, Rongling Wu

**Affiliations:** ^1^College of Science, Beijing Forestry University, Beijing, China; ^2^College of Biology and the Environment, Nanjing Forestry University, Nanjing, China; ^3^Center for Computational Biology, College of Biological Sciences and Technology, Beijing Forestry University, Beijing, China; ^4^Faculty of Science, Yamagata University, Yamagata, Japan; ^5^Departments of Public Health Sciences and Statistics, Center for Statistical Genetics, The Pennsylvania State University, Hershey, PA, United States

**Keywords:** trees growth, genetic architecture, quantitative trait loci (QTLs), nonlinear mixed mapping, multiphasic growth models

## Abstract

Trait covariation during multiphasic growth is of crucial significance to optimal survival and reproduction during the entire life cycle. However, current analyses are mainly focused on the study of individual traits, but exploring how genes determine trait interdependence spanning multiphasic growth processes remains challenging. In this study, we constructed a nonlinear mixed mapping framework to explore the genetic mechanisms that regulate multiphasic growth changes between two complex traits and used this framework to study stem diameter and stem height in forest trees. The multiphasic nonlinear mixed mapping framework was implemented in system mapping, by which several key quantitative trait loci were found to interpret the process and pattern of stem wood growth by regulating the ecological interactions of stem apical and lateral growth. We quantified the timing and pattern of the vegetative phase transition between independently regulated, temporally coordinated processes. Furthermore, we visualized the genetic machinery of significant loci, including genetic effects, genetic contribution analysis, and the regulatory relationship between these markers in the network structure. We validated the utility of the new mapping framework experimentally *via* computer simulations. The results may improve our understanding of the evolution of development in changing environments.

## Introduction

Mapping the genetic architecture of complex traits is a subject of long-standing interest and a formidable challenge in modern biology (Falconer et al., 1990; Mackay, 2001; Heslot et al., 2014). Quantitative trait locus (QTL) mapping is often performed to identify QTLs or causal genes associated with phenotypes of interest (Mackay et al., 2009), and has been successfully applied in many plant breeding programs (Collard and Mackill, 2008). Classic quantitative genetic mapping based on univariate analysis results serves as a simple method for comparing the genetic control of growth at different ages in a variety of organisms (Dieters et al., 1995; Suzanne, 2001). More powerful mapping strategies have been developed for the genetic mapping of complex traits by integrating infinite-dimensional models, random regression theory (Meyer, 1998), stochastic process theory (Pletcher and Geyer, 1999), and functional mapping and systems mapping (Wu and Lin, 2006; Gai et al., 2011; Sun and Wu, 2015).

Trait formation exhibits many distinct transitions from juvenile to adulthood and from the vegetative phase to the reproductive phase. Many attempts have been made to unveil the genetic mechanisms that control the growth and developmental transitions of organisms at different phases (Bond, 2000; Tang et al., 2018; Ahsan et al., 2019; Xing et al., 2020). Xu et al. (2016) integrated a multiphase growth equation into a framework of functional mapping to reveal how QTLs mediate the early and late stages of tree stem wood in different fashions. Fu et al. (2017) expanded the theory of systems mapping to characterize specific QTLs that mediate cooperation and competition between different traits. However, genetic control mechanisms underlying trait covariation across multiphase growth have rarely been explored.

In this study, we consider the fact that different phases of trait growth and development exhibit different growth characteristics. It is highly important to divide the phases of growth for the efficient utilization of resources. Phase changes are usually identified by modeling temporal patterns of growth and using mathematical equations from biological and statistical perspectives. As an example, we study two different but developmentally interdependent traits, primary height growth, and secondary radial growth, of forest trees. We take advantages of the classic Lotka–Volterra (LV) model (May, 1975), which was originally proposed to describe the ecological interaction of two species and explain the complexity problem of ecosystems. We defined a nonlinear governing equation (NGE) combining the two traits to describe not only the growth patterns of diameter and height but also their potential interaction pattern. We analyzed the genetic control of single-nucleotide polymorphisms (SNPs) with significant effects on the growth of complex phases, which were identified by the NGE model. On the basis of genetic effects, we established a regulatory network among significant markers to realize the comprehensive analysis of stem wood growth in multiple phases. This study could be beneficial for the management of forest plantations and the improvement of fast-growing forest varieties.

## Materials and Methods

### Mapping Materials

Xu et al. (2016) reported a genetic linkage mapping study on growth traits in a full-sib family of *Populus*. This family was generated by crossing an eastern cottonwood (*Populus deltoides*) clone I-69, introduced to China in the 1970s (Wu et al., 1992), as the female parent, and a Euramerican poplar (*P*. × *euramericana*) clone I-45 as the male parent. This cross is equivalent to a backcross at the species level. This family was planted in 1984 for 24 years in a uniform site at Zhangji Forest Farm, Xuzhou, Jiangsu, China. Supplementary Table S1 shows the average of annual temperature and annual precipitation at the experimental site from 1987 to 2010. Stem heights (defined as the length of the main stem from the stem–root connection to the tip) and stem diameters at breast height were measured for each tree at the end of each growing season. A part of this family (64 full-sib members) was genotyped, producing 156,362 good-quality SNPs distributed on 19 chromosomes. Of these SNPs, 94,591 were backcross-like testcross markers and 61,771 were F_2_-like intercross markers. For a testcross marker, one of the parents is heterozygous and the other is homozygous, and the intercross marker is derived from two heterozygous parents.

Stem growth in Populus trees experiences multiple phases of development including juvenile, mature, and senescence (Bond, 2000). In the study of Xu et al. (2016), only data from the first 14 years were considered, which include two possible phases. Because it is likely that tree growth experiences a distrain phase from year 14, a joint analysis of stem growth that spans24 years is essential to reveal the genetic architecture of phase change in stem wood growth trajectories. To do so, a more sophisticated model, as we will develop in this study, is needed.

### Multiphasic Growth Equation

Several classical nonlinear growth equations, such as those of Gompertz (Gompertz, 1825), Richards (Richards, 1959), Logistic (Verhulst, 1838), and Von Bertalanffy (Bertalanffy, 1957), describe the “S” shape of growth approximation. However, the growth of most organisms can actually be described as a composite form of multiple “S”-shaped phases because of seasonal fluctuations and differences in the growth rates of the components of organisms (Piantadosi, 1987). Koops proposed a multiphasic growth model, which is superior to single-phase growth in many studies and has been shown to more accurately estimate growth parameters (Koops, 1987; Grossman and Koops, 1988; Kwakkel et al., 1993). For example, Van der Klein et al. (2020) compared the monophasic, diphasic, and triphasic Gompertz and logistic models to describe the weight-age and gain-age functions of hens, and the results showed that the diphasic and triphasic Gompertz and logistic models yielded better fitting effects than the monophasic models. In addition, Treves et al. (2017) studied the multiphase growth pattern of the green alga Chlorella ohadii and metabolic changes during the growth phases. Multiphase analysis has proven to be a more accurate method for fitting biological data analysis and prediction.

According to the multiphasic growth view proposed by Koops (1987), the overall growth of height and diameter of poplar in the first 24 years is expressed as the sum of the growth function of two phases, juvenile and early adult (Xu et al., 2016), leading to a coupled nonlinear governing equation (NGE):


(1) $$\{\begin{array}{l} H\left(t\right)=H_{1}\left(t\right)+H_{2}\left(t\right)\\ D\left(t\right)=D_{1}\left(t\right)+D_{2}\left(t\right) \end{array},$$

where *H*(*t*) and *D*(*t*) represent the growth of quantitative traits of height and diameter of trees at age t; *H*_1_(*t*) and *D*_1_(*t*) represent growth during the juvenile phase; and *H*_2_(*t*) and *D*_2_(*t*) represent the growth in adulthood.

In nature, the interactive relationship between diameter and height can promote or hinder the growth of another trait. The synergistic effect of stem height and radial growth is conducive to the survival and reproduction of trees. However, when resources are in short supply, height growth and diameter growth achieve a trade-off of resource competition (Hulshof et al., 2015). In particular, it is significant and obvious during the juvenile stage of vegetative growth and nutrient accumulation (Fu et al., 2017). The governing relationship between diameter and height during the juvenile stage can be expressed as:


(2) $$\{\begin{array}{c} \frac{dH_{1}}{dt}=\alpha_{H}\left(1-\frac{H_{1}}{K_{H_{1}}}\right)H_{1}+\alpha_{H}\beta_{H\leftarrow D}H_{1}D_{1}\\ \frac{dD_{1}}{dt}=\alpha_{D}\left(1-\frac{D_{1}}{K_{D_{1}}}\right)D_{1}+\alpha_{D}\beta_{D\leftarrow H}D_{1}H_{1} \end{array},$$

where *H*_1_ and *D*_1_ represent the stem height and diameter of poplar in the first phase, which corresponds to juveniles. According to the form of the equation, the first phase can be divided into independent growth and interaction growth. α_*H*_ and α_*D*_ represent the independent growth rate of the first phase; *K*_*H*_1__ and *K*_*D*_1__ represent the asymptotic value of independent growth of two characters in the first phase; and β_*H*←*D*_ and β_*D*←*H*_ are dimensionless parameters used to describe the competitive or cooperative interaction between stem height and diameter.

However, when poplar reaches the maturity period of development, the growth patterns of diameter and height change, which can be described by growth equations without interacting parts (Xu et al., 2016). By evaluating the information criterion and fitting optimum of the equations through numerical experiments in Supplementary Table S2, we introduce the nonlinear governing equation (NGE), with *H*_2_(*t*) and *D*_2_(*t*) satisfying the following two equations:


(3) $$\{\begin{array}{l} H_{2}\left(t\right)=K_{H_{2}}\exp\;\left(-\exp\left(p_{H}-q_{H}t\right)\right)\\ D_{2}\left(t\right)=K_{D_{2}}\exp\;\left(-\exp\left(p_{D}-q_{D}t\right)\right) \end{array}$$

where *K*_*H*_2__ and *K*_*D*_2__ are the growth asymptotic values of adulthood height and diameter; *p*_*H*_ and *p*_*D*_ are related to the initial values of adulthood; and *q*_*H*_ and *q*_*D*_ represent the growth rate of adulthood.

The NGE can not only clarify the dynamic change rule of the growth of each character at different phases but also represent the governing rule between two interaction traits in the first phase.

### Modeling Framework of Multiphasic QTL Mapping

We consider the multiphasic growth of height and diameter as a whole according to the NGE and detect how QTLs controlled the overall growth curve from the perspective of system mapping, as well as the regulation of phase transition by QTLs (Gai et al., 2011; Sun and Wu, 2015). We design a model framework that takes *n* samples as the mapping population. The trait growth of each sample i was measured in a series of time points1, ⋯ , *T*, and *y*_1*i*_ = (*y*_1*i*_(1), ⋯ , *y*_1*i*_(*T*)) and *y*_2*i*_ = (*y*_2*i*_(1), ⋯ , *y*_2*i*_(*T*))(*i* = 1, ⋯ , *n*) represent phenotypic data of time-related traits 1 (height) and 2 (diameter), respectively. The composite growth characteristics composed of two traits approximately obey the bivariate normal distribution, where the time-dependent mean value and the symmetric covariance matrix (2*T* × 2*T*) are, respectively, expressed as


$$\begin{array}{l} \mu =\left(\mu_{1};\mu_{2}\right)=\left(\mu_{1}\left(1\right),\cdots ,\mu_{1}\left(T\right);\mu_{2}\left(1\right),\cdots ,\mu_{2}\left(T\right)\right)\\ \;\Sigma =\left(\begin{array}{cc} \Sigma_{1} & \;\Sigma_{12}\\ \Sigma_{21} & \;\Sigma_{2} \end{array}\right) \end{array}$$

The elements on the diagonal inΣ are the variance matrix for each trait, and the nondiagonal elements are the covariance matrix between a pair of traits. The joint density function of the two-dimensional normal vectors is of the form*f*(*y*_1_, *y*_2_; θ), where θ represents the growth parameters. The joint probability density function of n sample trees constitutes the likelihood function as follows:


(4) $$L_{0}=f\left(y_{11},y_{21};\theta \right)\cdots f\left(y_{1n},y_{2n};\theta \right)=\prod_{i=1}^{n}f\left(y_{1i},y_{2i};\theta \right).$$

It is assumed that the multiphasic growth of the two traits is controlled by a set of QTLs located on the linkage map. J kinds of genotypes are on the assumed QTL, and the time-dependent mean values of the samples differ among genotypes:


$$\mu_{j}=\left(\mu_{j1};\mu_{j2}\right)=\left(\mu_{j1}\left(1\right),\cdots ,\mu_{j1}\left(T\right);\mu_{j2}\left(1\right),\cdots ,\mu_{j2}\left(T\right)\right).$$

The joint density function of the genotype is denoted by *f*_*j*_(*y*_1_, *y*_2_; θ_*j*_)(*j* = 1, ⋯ , *J*). On the assumed specific QTL, the number of samples with genotype j is *n*_*j*_, satisfying $\overset{j}{\underset{j=1}{\sum }}n_{j}=n$. The mixed likelihood function is expressed as


(5) $$\begin{array}{l} L_{1}=\prod_{i=1}^{n_{1}}f_{1}\left(y_{1i},y_{2i};\theta_{1}\right)\cdots \prod_{i=1}^{n_{J}}f_{J}\left(y_{1i},y_{2i};\theta_{J}\right)\\ \;=\prod_{j=1}^{J}\prod_{i=1}^{n_{j}}f_{j}\left(y_{1i},y_{2i};\theta_{j}\right). \end{array}$$

According to the multiphasic growth function of the NGE, we can estimate the mean vector through a set of parameters (α_*jH*_, *K*_*j*_*H*__1__, β_*jH*←*D*_, α_*jD*_, *K*_*j*_*D*__1__, β_*jD*←*H*_, *K*_*j*_*H*__2__, *p*_*jH*_, *q*_*jH*_, *K*_*j*_*D*__2__, *p*_*jD*_, *q*_*jD*_)(*j* = 1, ⋯ , *J*) instead of directly estimating 2T mean values. By comparing the genotype-dependent differences in this parameter set, we can determine whether the QTL affects multiphasic growth.

In addition, we use a highly efficient structured antedependence (SAD) (1) statistical model, which was proposed by Zhao et al. (2005), to represent the longitudinal covariance matrix Σ. This method uses a few parameters to calculate the matrix with a complex structure, which offers the advantages of simplicity and flexibility and greatly improves the computational efficiency and statistical ability of the QTL detection model. The innovation variance and the first-order pre-dependent parameter are defined as $\gamma_{1}^{2},\;\gamma_{2}^{2\;}\;$and ϕ_1_, ϕ_2_, respectively, to structure the residual variance of trait k (k = 1,2) at time t:


(6a) $$Var\left(e_{k}\left(t\right)\right)=\frac{1-\phi_{k}^{2t}}{1-\phi_{k}^{2}}\gamma_{k}^{2}.$$

The covariance between *t*_1_ and *t*_2_ is expressed as


(6b) $$Cov\left(e_{k}\left(t_{1}\right),e_{k}\left(t_{2}\right)\right)=\phi_{k}^{t_{2}-t_{1}}\frac{1-\phi_{k}^{2t_{1}}}{1-\phi_{k}^{2}}\gamma_{k}^{2},t_{2}>t_{1}.$$

The fixed innovation variance between different time points is represented by parameter ρ, and the covariance between the two traits at different time points is expressed as


(6c) $$Cov\left(e_{1}\left(t_{1}\right),e_{2}\left(t_{2}\right)\right)=\frac{\phi_{2}^{t_{2}-t_{1}}-\phi_{1}^{t_{1}}\phi_{2}^{t_{2}}}{1-\phi_{1}\phi_{2}}\rho \gamma_{1}\gamma_{2},t_{2}>t_{1}.$$

In the calculation, we maintain that the parameters of the covariance matrix under different genotypes of *L*_0_ and *L*_1_ are consistent, which can improve the operation efficiency of the model with little loss of precision. The maximum likelihood estimation of parameters in the mean and covariance structure of the QTL detection model is performed by applying the expectation maximization (EM) algorithm (Dempster, 1977). The fourth-order Runge–Kutta algorithm is used to calculate the parametric solution of the NGE, and the Nelder–Mead simplex algorithm (Zhao et al., 2004) is used as the optimization method to estimate the parameters of the nonlinear equation and matrix structure, making the model more efficient.

### Hypothesis Testing

On the basis of the likelihood values *L*_0_ and *L*_1_, we detected the existence of QTLs that influence the multiphasic growth of traits by calculating the likelihood ratio statistic. By comparing the genotypic correlation differences within the parameter set, the existence of QTLs affecting the multiphasic growth of stem height and diameter was determined:


(7) $$\begin{array}{l} H_{0}:\\ \left(\alpha_{jH},K_{jH_{1}},\beta_{jH\leftarrow D},\alpha_{jD},K_{jD_{1}},\beta_{jD\leftarrow H},K_{jH_{2}},p_{jH},q_{jH},K_{jD_{2}},p_{jD},q_{jD}\right)\\ =\left(\alpha_{H},K_{H_{1}},\beta_{H\leftarrow D},\alpha_{D},K_{D_{1}},\beta_{D\leftarrow H},K_{H_{2}},p_{H},q_{H},K_{D_{2}},p_{D},q_{D}\right)\\ forj=1,\cdots ,J \end{array}$$

*H*_1_: At least one of the above equalities does not hold true.

The null hypothesis means that growth is consistent across different genotypes, which could be described by a set of NGE parameters. The alternative hypothesis is that there are genotype differences in the NGE parameters. The form of the likelihood ratio statistic is as follows:


(8) $$LR=2\log\left(\frac{L_{1}}{L_{0}}\right).$$

LR approximately obeys theχ^2^distribution and its degree of freedom is the difference in the number of model parameters between *H*_0_ and *H*_1_.

The rejection domain of the likelihood ratio statistic LR is taken as *W* = {*LR* ≥ *c*}, where the critical value c satisfies:


$$P_{\theta }(LR\geq c)\leq \alpha .$$

At the set test level α, if LR belongs to the rejection domain, we reject the null hypothesis *H*_0_ and accept the alternative hypothesis *H*_1_, which indicates that at this significance level, differences exist in the multiphasic growth of different genotypes of this marker. The *p*-values for hypothesis tests are converted by LRs and compared with the critical thresholds. We implemented stringent multiple testing procedures to control the false positive rate. On the one hand, we performed Bonferroni correction to adjust the critical value to ensure more rigorous results. On the other hand, a false discovery rate (FDR) correction based on the Benjamini and Hochberg method was performed on each *p*-value to control the proportion of false positives within a certain range.

We can further examine how QTLs control the multiphasic growth of the two traits and the characteristics in different phases. Based on the following hypotheses, we can detect whether the QTL regulates the interaction growth of stem height and diameter in the first phase:


$$\begin{array}{l} H_{0}:\left(\alpha_{jH},K_{jH_{1}},\beta_{jH\leftarrow D},\alpha_{jD},K_{jD_{1}},\beta_{jD\leftarrow H}\right)\\ =\left(\alpha_{H},K_{H_{1}},\beta_{H\leftarrow D},\alpha_{D},K_{D_{1}},\beta_{D\leftarrow H}\right)\\ H_{1}:\left(\alpha_{jH},K_{jH_{1}},\beta_{jH\leftarrow D},\alpha_{jD},K_{jD_{1}},\beta_{jD\leftarrow H}\right)\\ \neq \left(\alpha_{H},K_{H_{1}},\beta_{H\leftarrow D},\alpha_{D},K_{D_{1}},\beta_{D\leftarrow H}\right)forj=1,\cdots ,J, \end{array}$$

and whether the QTL controls the growth of the two traits in the second phase by formulating the following hypotheses:


$$\begin{array}{l} H_{0}:\left(K_{jk_{2}},p_{jk},q_{jk}\right)=\left(K_{k_{2}},p_{k},q_{k}\right)\\ H_{1}:\left(K_{jk_{2}},p_{jk},q_{jk}\right)\\ \neq \left(K_{k_{2}},p_{k},q_{k}\right)forj=1,\cdots ,J;k=H\;or\;D. \end{array}$$

According to the biological significance of the parameter, we can also test whether QTLs control the asymptotic growths *K*_*H*_1__ and *K*_*D*_1__ of the independent growth of the two traits in the first phase, the total asymptotic growth with respect to the interaction, and the asymptotic growths *K*_*H*_2__ and *K*_*D*_2__ in the second phase. In addition, the independent growth rate of the first phases α_*H*_ and α_*D*_, and the interaction relationships β_*H*←*D*_ and β_*D*←*H*_ of the first phase can also be tested. The initial growth values *p*_*H*_ and *p*_*D*_ and growth rates *q*_*H*_ and *q*_*D*_ of the second phase can also be tested. The phase transition time was determined by examining the differences in parameters with respect to genotypes.

## Results

### Fitting Multiphasic Growth Trajectories

**Figure 1:**
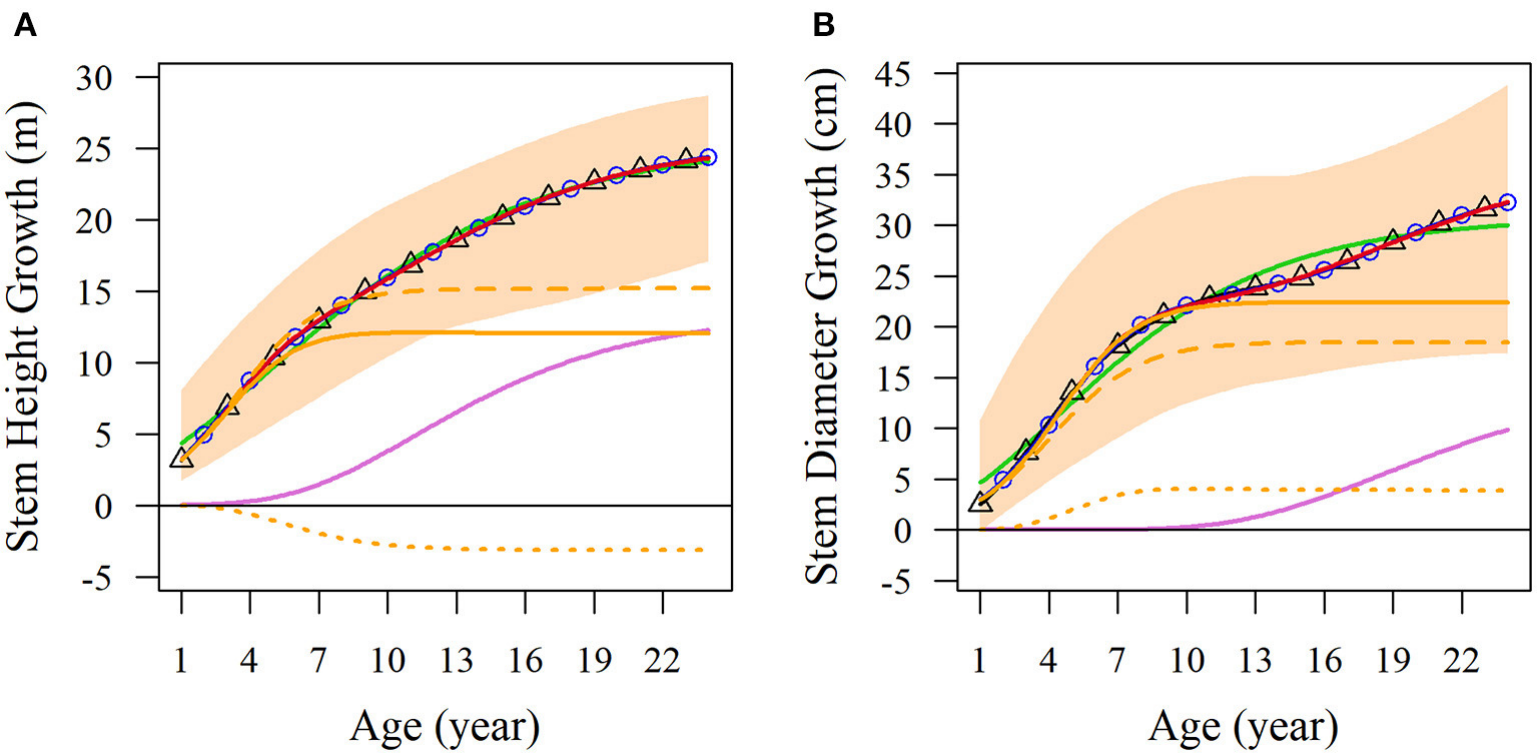
Average growth trajectories of Populus growth. Observed phenotypic ranges of **(A)** stem height and **(B)** stem diameter of all the samples are indicated by light orange shading. Average curves of the two traits are fitted with the monophasic equation (green lines), diphasic equation (black lines with triangles), triphasic equation (blue lines with circles), and NGE (red lines). NGE growth curves are divided into the first phase of orange curves and the second phase of light purple curves. In the first phase, the curves are divided into broken lines to represent independent growth, and dotted lines to represent the interaction growth of the two characters.

We used the NGE (Equation 1) to fit the diameter and height growth data compared with monophasic, diphasic, and triphasic logistic models. In Figure 1, the fitting effects of the logistic models are good during the early stage of tree growth (approximately 7 years), but the NGE model has a better fitting effect during the later growth stage. This indicates that the whole growth trajectory of trees in different periods is changing. We compared the performances of these models by calculating the Akaike information criterion (AIC), Bayesian information criterion (BIC), Schwarz criterion, and adjusted *R*^2^ (Table 1). As shown, the NGE performs better than the other growth equations, suggesting that it is crucial to consider the impact of interactive traits at different stages of growth. We also fitted the growth of 66 samples with the NGE, and the growth of both height and diameter for each hybrid showed excellent goodness of fit to the NGE (*R*^2^ > 0.976; Supplementary Figures S1, S2). The residual errors of the growth data are distributed randomly over the predicted values (Supplementary Figure S3), suggesting that the NGE model is quite robust.

**Table 1:** Estimated parameters of fitting and evaluation information.

**Monophasic**		**Diphasic**		**Triphasic**		**Diphasic (NGE)**	
**Height**	**Diameter**	**Height**	**Diameter**	**Height**	**Diameter**	**Height**	**Diameter**
*p* = 0.7198	*p* = 0.8207	*p*_1_ = 2.5808	*p*_1_ = 5.0151	*p*_1_ = 0.5617	*p*_1_ = -8.2512	α_*H*_ = 0.5647	α_*D*_ = 0.5399
*q* = 0.1497	*q* = 0.1853	*q*_1_ = 0.1925	*q*_1_ = 0.2677	*q*_1_ = 0.1321	*q*_1_ = -0.8430	*k*_*H*_1__ = 14.9231	*k*_*H*_1__ = 19.9831
*K* = 25.5181	*K* = 30.8222	*K*_1_ = 9.8744	*K*_1_ = 9.5220	*K*_1_ = 7.6007	*K*_1_ = 1.6566	β_*H*←*D*_ = -0.0092	β_*D*←*H*_ = 0.0059
		*p*_2_ = 0.8054	*p*_2_ = 1.1539	*p*_2_ = 2.5714	*p*_2_ = 4.8608	*p*_*H*_ =1.7521	*p*_*D*_ = 2.5552
		*q*_2_ = 0.3326	*q*_2_ = 0.3321	*q*_2_ = 0.1960	*q*_2_ = 0.2574	*q*_*H*_ = 0.1755	*q*_*D*_ = 0.1293
		*K*_2_ = 15.7204	*K*_2_ = 24.7699	*K*_2_ = 7.7947	*K*_2_ = 9.5961	*K*_*H*_2__ = 16.1135	*K*_*D*_2__ = 18.6017
				*p*_3_ = 1.0558	*p*_3_ = 1.3770		
				*q*_3_ = 0.4139	*q*_3_ = 0.3320		
				*K*_3_ = 10.4079	*K*_3_ = 24.9187		
*adj*.*R*^2^= 0.9727		*adj*.*R*^2^= 0.9887		*adj*.*R*^2^= 0.9887		*adj*.*R*^2^= 0.9982	
RSD = 11.8756		RSD = 7.2444		RSD = 6.8235		RSD = 3.0006	
AIC = 13.7405		AIC = 12.1907		AIC = 12.0881		AIC = 5.2173	
BIC = 13.9395		BIC = 12.5888		BIC = 12.6853		BIC = 5.7972	

*Monophasic, diphasic, triphasic, and nonlinear governing equation (NGE) models were used to fit the average growth of stem height and stem diameter of Populus*.

Obviously, the fitting results of the NGE can be divided into two parts; the first stage of which includes potential independent growth and interaction growth. Figure 1 shows the fitting results of the average growth curve. The first growth phase of stem height reached its asymptotic value at 7 years of age, and the age at which the asymptotic value of independent growth was reached was slightly later than that of the total growth of the first phase. Moreover, we found that in the first phase, the growth of stem height competed with diameter, leading to independent growth surpassing the total growth. In addition, the stem height growth of the second phase begins early, so the two phases overlap by a considerable period of time (Figure 1A). On the other hand, the stem diameter growth reached the asymptotic value of the first phase on the 10th year. The overall growth of stem diameter during this phase was higher than the independent growth curve, indicating that the growth of stem diameter was promoted by stem height. In addition, the second phase of diameter growth started later than that of the stem height and was far from asymptotic over the 24 years of the observed data (Figure 1B).

### QTL Detection Based on the NGE

**Figure 2:**
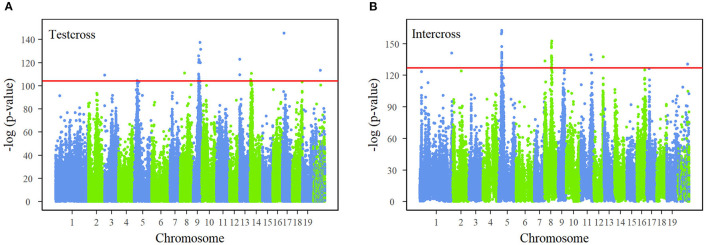
Manhattan plots of *p*-values across 19 chromosomes of the Populus genome. Test statistic values of single-nucleotide polymorphisms (SNPs) were calculated by the system mapping of 24-yr growth curves of stem height and diameter. The *p*-values are obtained after false discovery rate (FDR) correction. Red horizontal lines are the critical thresholds at the 10^−40^and 10^−50^ significance levels for **(A)** test cross SNPs and **(B)** intercross SNPs obtained by Bonferroni correction.

The joint process of multiphasic growth is regarded as a whole system, containing interactions between traits in the first phase and growth characteristics. By regressing the genotype-related growth trajectory of stem diameter and stem height, the differences in genotypes are expressed in the NGE parameters (α_*jH*_, *K*_*j*_*H*__1__, β_*jH*←*D*_, α_*jD*_, *K*_*j*_*D*__1__, β_*jD*←*H*_, *K*_*j*_*H*__2__, *p*_*jH*_, *q*_*jH*_, *K*_*j*_*D*__2__, *p*_*jD*_, *q*_*jD*_). The description of how a QTL affects the multiphasic growth and intrinsic interaction of traits can be characterized from genome-wide information based on a series of hypothetical tests. We performed FDR correction on the *p*-value results of all the SNPs and determined the critical thresholds as 10^−40^ and 10^−50^ after Bonferroni correction for testcross SNPs and intercross SNPs, which are strict detection levels. The Manhattan plots of the corrected *p*-values derived from test statistic values are shown in Figure 2. Our NGE-based mapping model identified 26 significant testcross SNPs and 39 intercross SNPs sporadically distributed over the genome, and 74% of significant SNPs were within candidate genes with known growth-related functions. For example, SNP 48,502 is within the *GATA12* gene, which is a member of the GATA family of transcription factors. A set of significant SNPs are closely distributed in the same region of chromosomes, such as chromosomes 5, 8, and 9, residing within the same gene. For instance, SNPs 79,620, 79,624, and 79,625 are highly linked on chromosome 8, located within the region of the *OASA1* gene, which encodes the synthesis of anthranilate synthase. Supplementary Table S3 presents basic information about all the significant SNPs, such as their chromosomal and physical locations, segregation types, allele types, *p*-values, and annotated candidate genes. Supplementary Table S4 displays specific genotype parameters of the significant SNPs. Supplementary Figure S4 depicts the genetic effect curves of the significant SNPs, showing the contribution to stem height and diameter growth. The heritability of stem height growth and stem diameter growth at different ages explained by each of the 65 significant SNPs is shown in Supplementary Figure S5.

### Genetic Architecture Analysis of Multiphasic Growth QTLs

**Figure 3:**
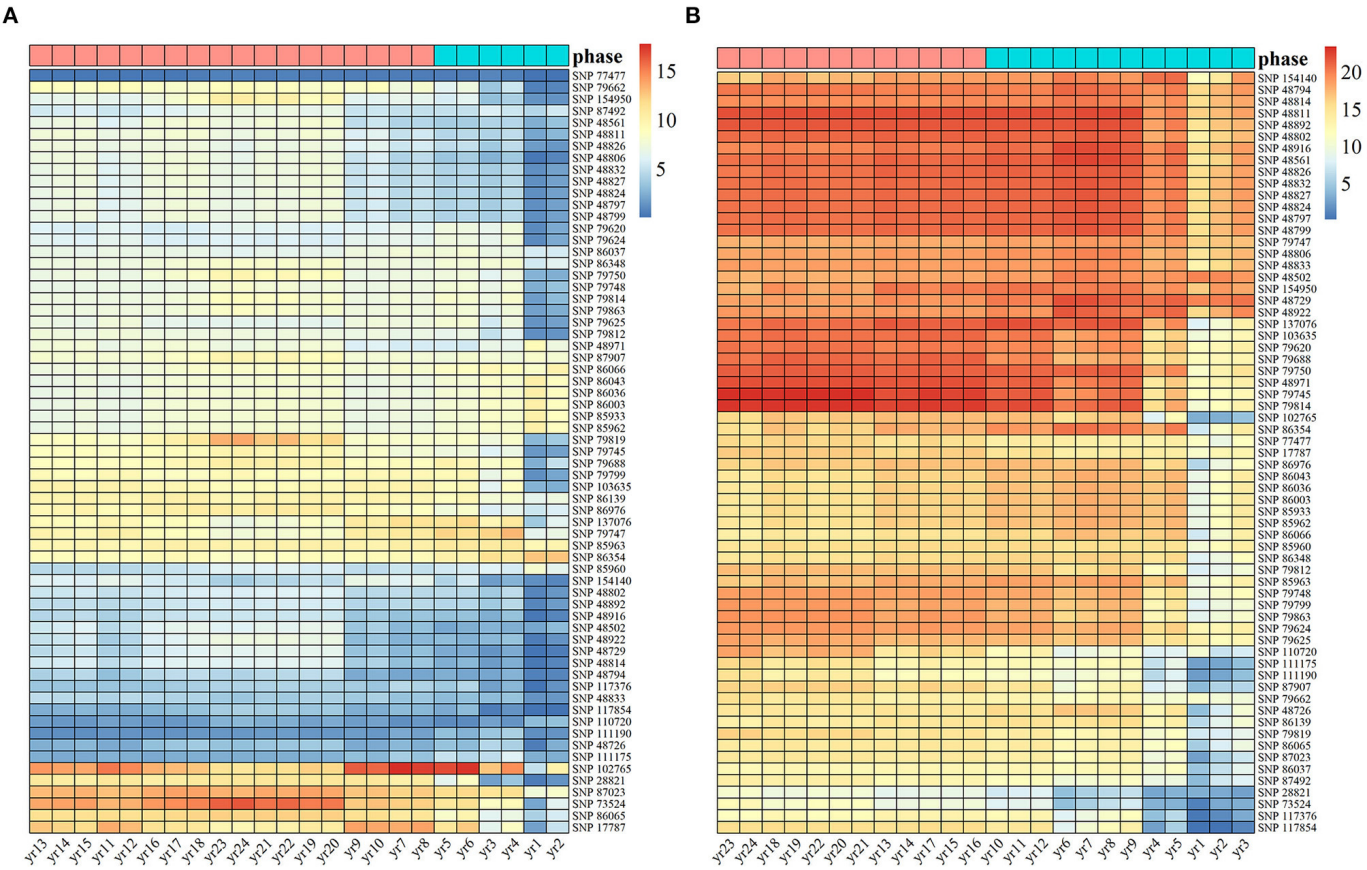
Heat maps of heritability are explained by 65 significant SNPs of Populus. The temporal patterns of heritability for **(A)** stem height and **(B)** stem diameter at 24 years were clustered into two phases, with blue representing the first phase and pink representing the second phase.

Based on the 65 identified SNPs with significant effects on multiphasic growth, we estimated genotype-dependent growth parameters for two different phases and calculated the phenotypic variation explained (PVE) for 24 years by quantifying the dynamic genetic contribution of markers to growth. The PVE of these loci for stem height growth was significantly smaller than the PVE for diameter. A basic hierarchical cluster analysis was performed for the PVE of all significant SNPs over time, which was divided into two stages and represented by tags with different colors. Of the observed 24 years of growth, the first phase of stem height growth was the first 6 years, and the remaining 18 years belonged to the second phase. The first phase of diameter growth was the first 12 years, and years 12–24 represented the second phase (Figure 3). Compared with the diameter, the first phase of stem height growth was shorter, which is approximately consistent with the phase estimation results of the NGE growth curve shown in Figure 1 and which further suggests the validity of the NGE analysis.

Among the significant loci, the PVE of the same SNP for the two traits was different. For example, the genetic contribution of SNPs 17,787, 86,065, 73,524, 87,023, 28,821, and 102,765 to the growth of stem height was relatively substantial, while the genetic contribution of SNPs P28821 and 73,524 to the growth of stem diameter was the lowest among all the SNPs. In addition, SNP 77,477, with a low genetic contribution to stem height, had a PVE of 10% in diameter. The genetic contribution of some SNPs, such as 28,821 and 73,524, is mainly expressed in the growth of one trait. However, some SNPs, such as 17,787 and 102,765, make strong genetic contributions to both stem diameter and stem height, indicating that the same significant SNP affects the growth of tree height and diameter in different ways, thus revealing the pleiotropy of genes.

**Figure 4:**
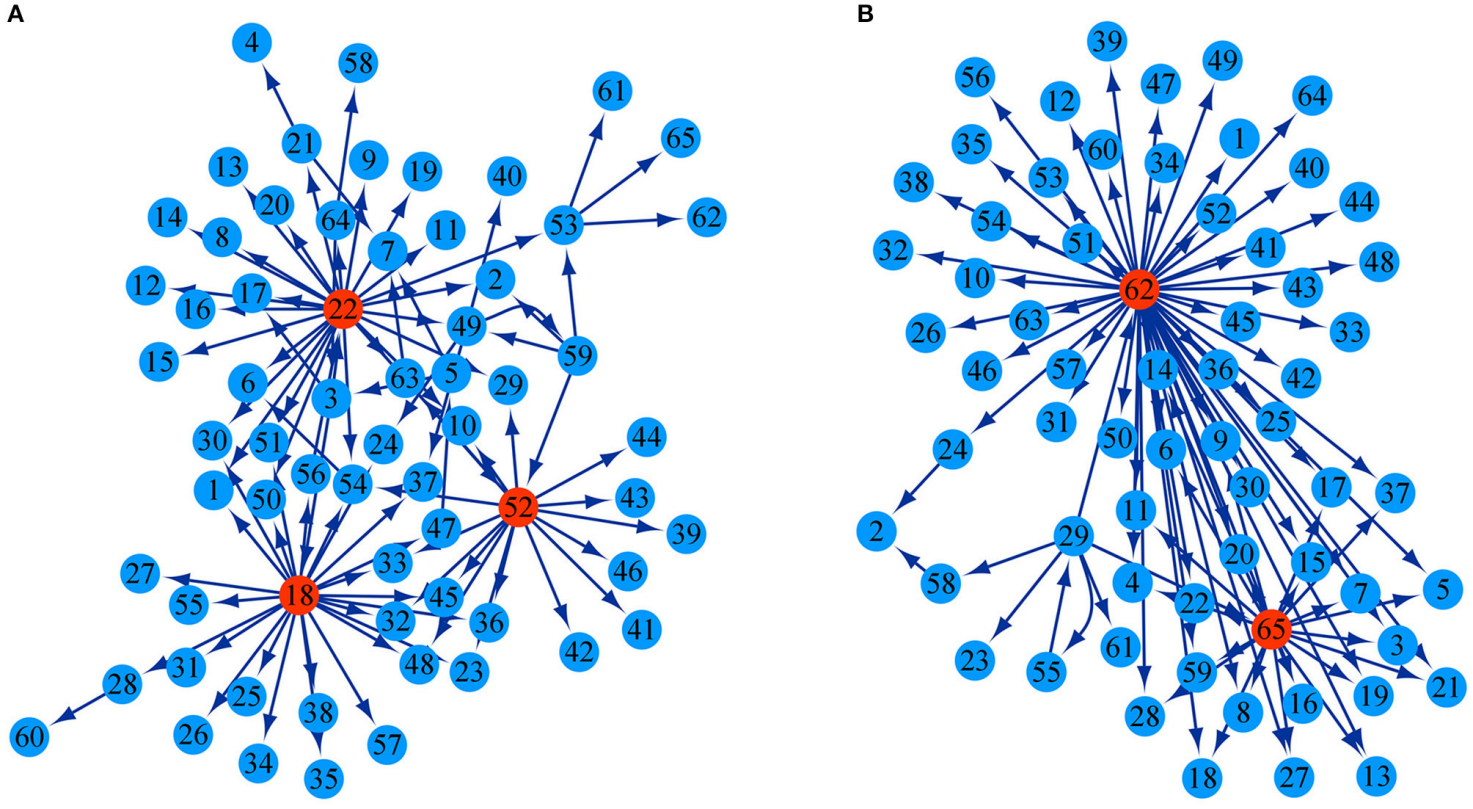
Genetic regulatory networks are generated through genetic effects. **(A)** Genetic network of 65 significant SNPs with respect to the growth trajectory of stem height. **(B)** Genetic network of 65 significant SNPs with respect to the growth trajectory of stem diameter. The arrow indicates the direction in which one SNP activates or inhibits another SNP. Highlight circles represent the key QTLs.

The growth of stem height and diameter is determined jointly by genetic effects of 26 testcross SNPs and 39 intercross SNPs as well as epistatic effects among them. According to the potential differential relationship of significant SNPs, we established a genetic network to characterize how these SNPs interact with each other to regulate the growth in diameter and height. In both the height and diameter network structures, most SNPs receive directional epistasis from other SNPs; they are activated or inhibited by other SNPs and play passive roles. Only a few significant SNPs called key QTLs were found to be in the pivotal dominant position of regulation in the genetic network, which had a strong potential to influence other QTLs with major outward links. Three SNPs, intercross SNP 48,833, intercross SNP 48,971, and testcross SNP 86,976, were the key QTLs in the stem height genetic network, and the directed links from them accounted for 27.17, 28.26, and 17.39% of all pairwise links, respectively (Figure 4A). The key QTLs in the stem diameter genetic network was testcross SNP 117,854 and intercross SNP 154,950, and links from the two directions accounted for 64.77 and 25% of the pairwise links, respectively (Figure 4B). Although these 65 significant SNPs jointly regulate the overall growth of diameter and height, they serve different roles in the structural networks for the growth of the two traits. The regulation relationship among SNPs is not singular in the network structure. For example, in the stem height network, there is reciprocal activation between SNP 86,139 and SNP 111,175. We discern a regulatory loop: SNP 48,833→SNP 86,065→SNP 48,726→SNP 48,502→SNP 48,833 in which one SNP regulates other SNPs but is influenced by another SNP. Details of the SNPs corresponding to each number are given in Supplementary Table S2.

### Genetic Control of Multiphasic Growth and Transition

The advantage of our NGE mapping approach lies in its capacity to analyze the genetic control of SNPs with significant effects on multiphasic growth, which can be tested by hypotheses based on related growth parameters. Here, we chose the representative testcross SNP 137,076 to interpret its genetic influence on growth in different phases of diameter and height. Both genotypes AG and AA in SNP 137,076 show that the growth of stem diameter benefits from stem height, and that stem height growth is deleteriously affected by diameter growth. This can be found from a similar form of overall growth and independent growth for stem height but greater overall growth relative to independent growth for stem diameter. The phase transition time of stem height occurs earlier than that of stem diameter. The two genotypes at this locus have similar control over the multiphasic growth of the two traits but different patterns in the growth details.

**Figure 5:**
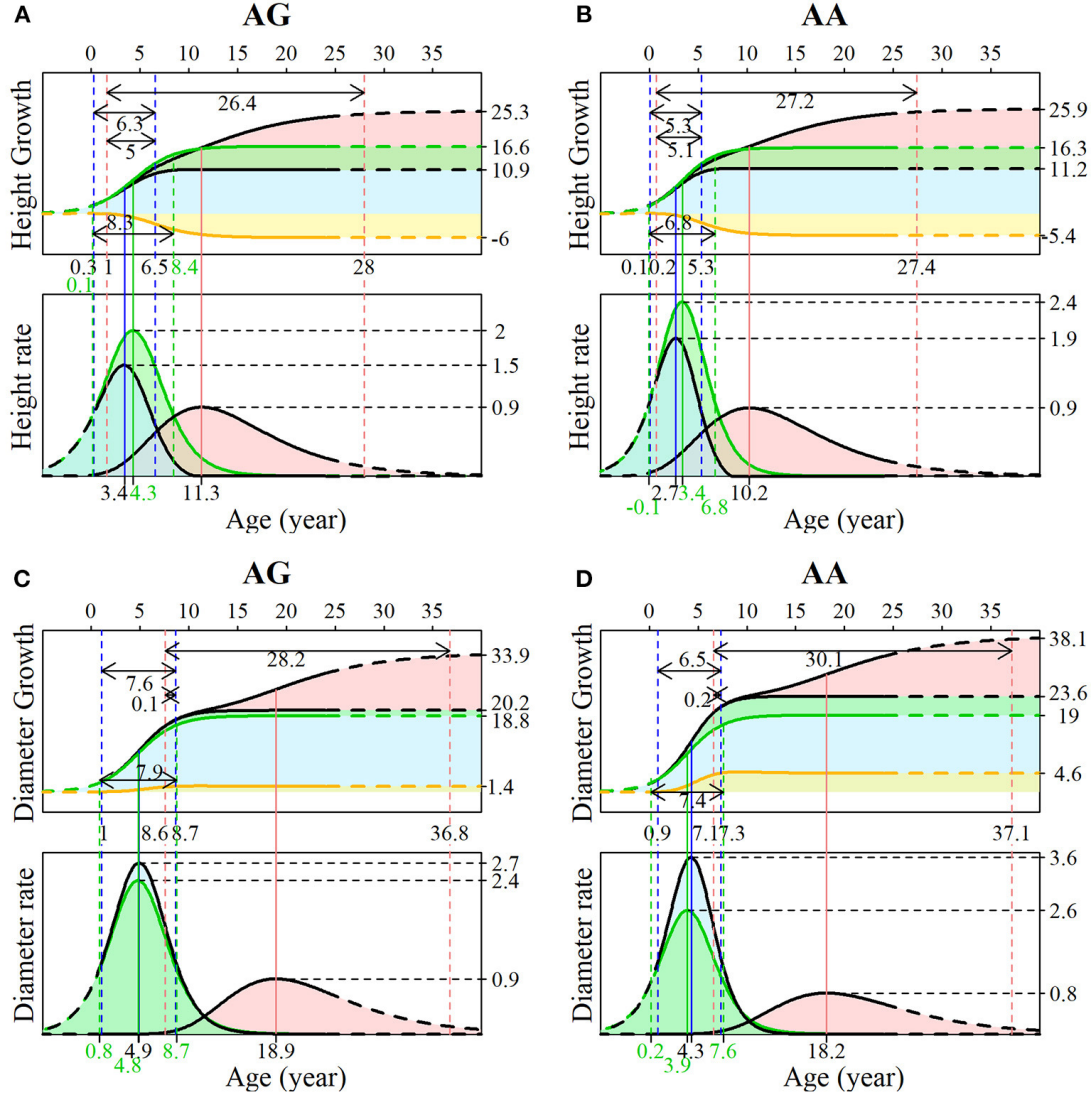
Genotypic growth curves and rate curves of stem **(A,B)** height and **(C,D)** diameter. The growth is explained by SNP 137076 with two genotypes, AG and AA. For each image, the upper panel is the growth curve, and the lower panel is the rate curve. The blue area represents the first phase of growth, where green represents the independent growth and yellow represents the interacting growth. The pink areas indicate the second phase of growth.

As shown in Figures 5A,B, during the juvenile phase, genotype AG had an extended period of duration of height growth compared with genotype AA (6.3 vs.5.3), and the difference was more pronounced for independent growth (8.3 vs. 6.8). Genotype AA has a larger asymptotic value (11.2 vs. 10.9) but a smaller asymptotic value for independent growth than genotype AG (16.3 vs. 16.6); thus, the latter suffers stronger inhibition (−6 vs. −5.4). The duration for the early adult phase of genotype AG was shorter (26.4 vs. 27.2), and the overlapping transition time between the two phases was also slightly shorter (5 vs. 5.1). According to the growth rate curve, the juvenile phase maximum growth rate of genotype AA was much larger than that of genotype AG (2.4 vs. 2), and there was a similar difference in the independent growth rate in this phase (3.6 vs. 2.7). The value of the early adult phase maximum growth rate was the same for both genotypes. As expected, the maximum growth rate and duration are determinants of overall growth. Different patterns in the onset, offset, duration, and maximum growth rate were found between the two genotypes for diameter growth (Figures 5C,D), suggesting that this SNP significantly affects not only the timing of growth but also the development between traits.

**Figure 6:**
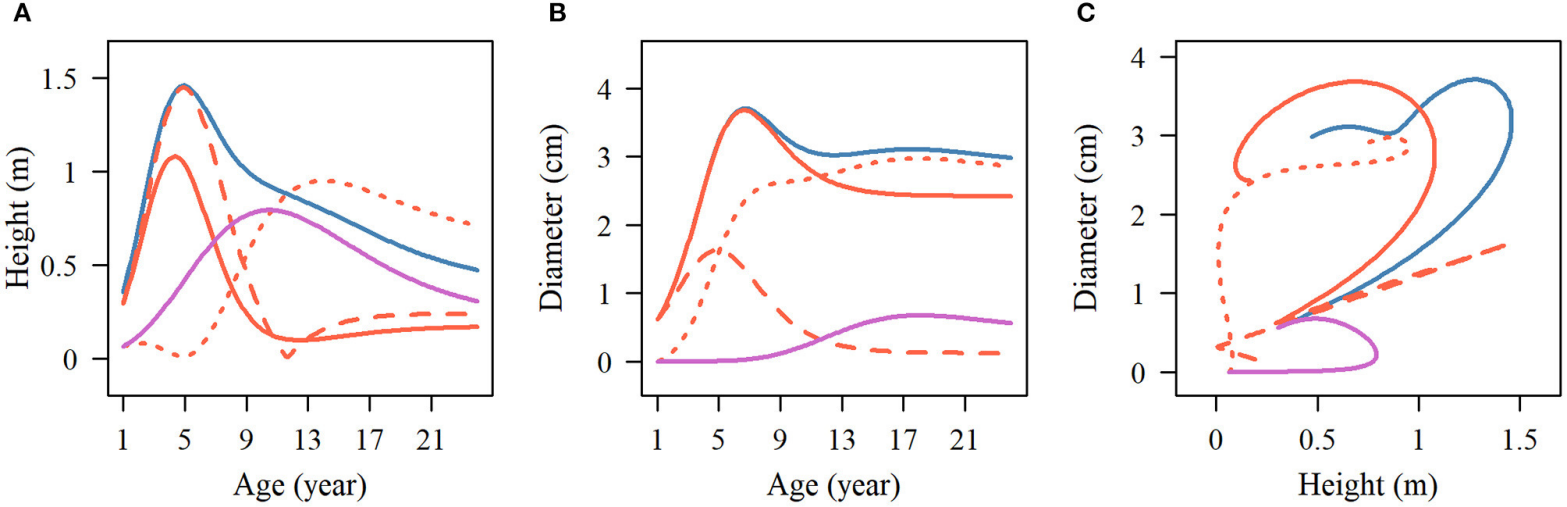
Temporal pattern of the pleiotropic effect on stem height and diameter. The multiphasic genetic effects of stem **(A)** height and **(B)** diameter, and the dynamic relationship of each effect on the two growth traits **(C)** at SNP 137076. The blue solid line represents the total effect of multiphasic growth, the red solid line displays the effect of the first phase, and the purple solid line denotes the effect of the second phase. The first phase effect curve was divided into independent (red broken line) and interaction (red dotted line) curves.

The influence of SNP 137,076 on the growth of the two traits is reflected in the temporal variation of the genetic effect, which also shows the characteristics of periodic change consistent with the growth curves. For stem height, the net genetic effect increases during the first 6 years and then decreases. During the juvenile phase, the overall genetic effect and the independent growth genetic effect show similar change patterns to the net effect, and then they level off at 12 years of age. The effect value of the independent growth is greater than that of the overall growth effect. The change in the interaction genetic effect shows a lag, beginning to rise at 5 years of age, and slowly declines until it stabilizes 12 years later. The genetic effect curve of the early adult phase gradually increased during years 1–12 and then decreased, which was related to the early growth of the second phase of stem height (Figure 6A). We analyzed a similar trend of genetic curves for diameter growth (Figure 6B). The pleiotropic effect between stem height and the diameter of SNP 137,076 is obvious with respect to the net genetic effect and juvenile phase genetic effect, but in the second phase, the curve shows two approximately flat trends, indicating that there is no obvious correlation between the growth of diameter and height in this phase. Thus, it is reasonable to describe the growth of the two traits without interaction in the second phase in the NGE (Figure 6C).

### Computer Simulation

To verify further the statistical characteristics and mapping accuracy of the NGE, we conducted a simulation analysis based on the real growth data of tree diameter and height over 24 years under different sample size and heritability conditions. Among them, the sample size is the number of simulated samples; namely, n = 66 (the same as the real example), 100, and 200. Heritability is the proportion of genetic variance in the simulated phenotypic variance, and two levels of *H*^2^are utilized: 0.05 and 0.1. It is assumed that the growth of the two traits of trees is regulated by a set of QTLs in the genome. The simulation scale is 1,000 testcross genetic markers, and the phenotypic data are determined by one of the QTLs. In each simulation case, the proportion of simulation iterations of meaningful QTLs screened out of 100 repeated simulation experiments is the mapping accuracy (power), which is calculated and shown in Table 2. In the analysis of the multiphasic growth of tree traits, the mapping accuracy of QTL detection based on the NGE exceeded 0.49, and the accuracy of simulation increased with heritability and sample size. With a heritability of *H*^2^= 0.05, although the accuracy was only 0.49 when the sample size was 66, it significantly improved to 0.82 when the size was increased to 100. When the sample size was 200, the accuracy of the two heritability levels reached 1. The results show that the NGE achieves good accuracy in selecting QTLs for multiphasic growth and is suitable for large sample size data analysis.

**Table 2:** Mapping accuracy (power) and false positive probability (FPR) of quantitative trait loci (QTLs) based on the NGE.

	***H***^**2**^ **= 0.05**			***H***^**2**^ **= 0.10**		
	***n* = 66**	***n* = 100**	***n* = 200**	***n* = 66**	***n* = 100**	***n* = 200**
Power	0.49	0.82	1.00	0.98	0.99	1.00
FPR	0.10	0.09	0.02	0.11	0.11	0.06

*The experiments simulated 66 (the same as the real example), 100, and 200 samples under heritability levels of 0, 0.05, and 0.1. The accuracy of positioning and FPR were evaluated by computer simulation*.

On the other hand, in the absence of QTL expression, the same genetic sample sizes of *n* = 66 (the same as the real example), 100, and 200 and heritability levels of *H*^2^= 0.05 and 0.1 are simulated with 1,000 genetic markers and phenotypic data. At this point, the proportion of the simulation times of QTLs screened out to the number of 100 simulations is the false positive probability. The false positive rate (FPR) of QTL detection based on the NGE is small (< 0.11), indicating that the NGE has reasonably high specificity even under the conditions of large sample size and heritability (Table 2).

**Figure 7:**
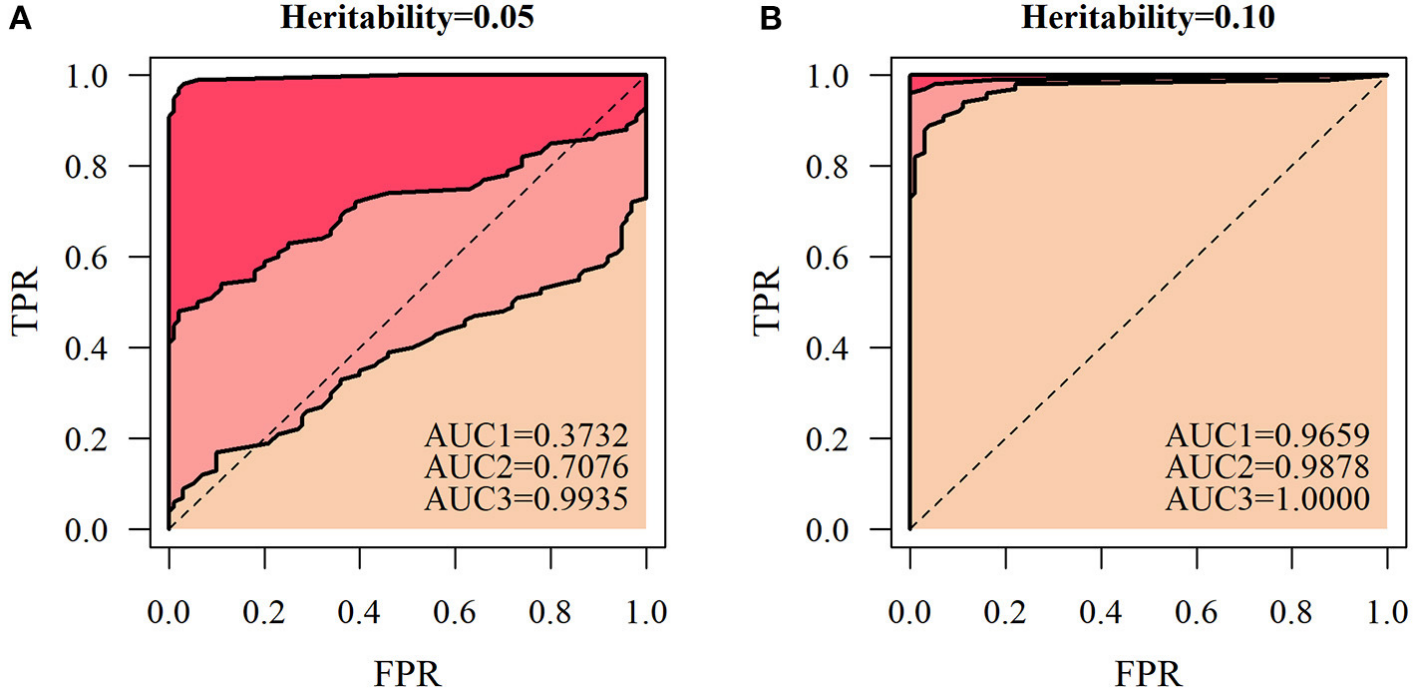
Receiver operating characteristic (ROC) curves of the heritability levels. Let the accuracy of QTL mapping be the ordinate, and the probability of false positives be the abscissa at heritability levels of **(A)** 0.05 and **(B)** 0.1 under the three cases of genetic sample sizes of 66, 100, and 200, respectively. Then, AUC1 represents the area under the ROC curve with a sample size of 66, AUC2 represents the area under the ROC curve with a sample size of 100, and AUC3 represents the area under the ROC curve with a sample size of 200.

According to the QTL mapping accuracy and false positive probability in a series of different thresholds, we express ROC curves for different simulated sample sizes. The area under the ROC curve (AUC) was calculated to assess the accuracy of QTL mapping of the NGE (Figure 7). Under several different simulation conditions, the AUC value was less than 0.5 only when *H*^2^= 0.05 and *n* = 66, and other simulation results had high QTL detection significance. At the heritability level of 0.1, the AUCs of the simulated quantities of the three scales were all relatively high (>0.9659). At a certain heritability level, the accuracy of QTL mapping based on the NGE is affected by the number of genetic samples, indicating that the model is more suitable for QTL detection with large sample size.

We perform 100 simulation calculations based on the NGE parameters of the two genotypes of a significant QTL at the heritability levels of *H*^2^ = 0.05 and 0.1, with sample sizes of *n* = 66, 100, and 200. The two genotypes are expressed as AA and Aa. The maximum likelihood estimation of NGE parameters (α_*jH*_, *K*_*j*_*H*__1__, β_*jH*←*D*_, α_*jD*_, *K*_*j*_*D*__1__, β_*jD*←*H*_, *K*_*j*_*H*__2__, *p*_*jH*_, *q*_*jH*_, *K*_*j*_*D*__2__, *p*_*jD*_, *q*_*jD*_) of simulated data is obtained by the fourth-order Runge–Kutta, EM, and Nelder–Mead algorithms. The parameter estimation results are shown in Table 3. Numerically, there are some differences between the estimated parameters and the real parameters. However, with the increase in genetic heritability and sample size, the precision of parameter estimation improves, the standard deviation of various parameters declines, and the simulation effec t becomes more stable.

**Table 3:** Parameter estimation of NGE.

	**True**		***H***^**2**^ **= 0.05**						***H***^**2**^ **= 0.10**					
			***n*** **= 66**		***n*** **= 100**		***n*** **= 200**		***n*** **= 66**		***n*** **= 100**		***n*** **= 200**	
	**AA**	**Aa**	**AA**	**Aa**	**AA**	**Aa**	**AA**	**Aa**	**AA**	**Aa**	**AA**	**Aa**	**AA**	**Aa**
α_*H*_	0.4816	0.5880	0.6689 (0.6401)	0.5827 (0.2164)	0.5506 (0.1955)	0.5553 (0.1053)	0.5366 (0.2111)	0.5424 (0.0659)	0.4663 (0.1314)	0.5086 (0.0546)	0.4640 (0.1171)	0.5296 (0.0643)	0.4629 (0.0635)	0.5268 (0.0389)
*K*_*H*1_	16.6452	16.3061	23.6395 (10.1328)	22.7284 (6.8697)	22.7374 (7.8829)	22.2026 (4.5249)	21.8139 (5.8606)	21.7824 (5.2260)	23.4571 (8.4108)	23.3385 (6.8279)	21.7667 (6.0535)	21.1651 (5.0928)	20.5583 (4.0174)	20.6019 (3.7874)
β_*H*←*D*_	-0.0085	-0.0082	-0.0215 (0.0230)	-0.0126 (0.0123)	-0.0182 (0.0141)	-0.0131 (0.0072)	-0.0182 (0.0102)	-0.0119 (0.0054)	-0.0179 (0.0099)	-0.0133 (0.0059)	-0.0177 (0.0079)	-0.0126 (0.0062)	-0.0189 (0.0057)	-0.0121 (0.0040)
α_*D*_	0.5057	0.5387	0.2934 (0.2643)	0.3443 (0.1301)	0.3164 (0.1974)	0.3489 (0.1023)	0.2961 (0.1268)	0.3474 (0.0862)	0.2762 (0.1528)	0.3762 (0.0976)	0.3114 (0.1146)	0.3779 (0.0788)	0.2940 (0.0950)	0.3815 (0.0563)
*K*_*D*_1__	18.8163	18.9900	13.6369 (7.9672)	16.5536 (6.1950)	14.8980 (5.2775)	16.5682 (3.7728)	15.1897 (3.8673)	16.1873 (3.2860)	13.4869 (5.2724)	15.1540 (4.0220)	14.4949 (4.0839)	15.5332 (3.4592)	14.8102 (3.5746)	15.7639 (2.6413)
β_*D*←*H*_	0.0034	0.0120	0.0140 (0.0245)	0.0166 (0.0130)	0.0113 (0.0120)	0.0154 (0.0078)	0.0127 (0.0085)	0.0165 (3.2860)	0.0171 (0.0145)	0.0216 (0.0109)	0.0157 (0.0116)	0.0204 (0.0091)	0.0161 (0.0096)	0.0200 (0.0073)
*P*_*H*_	1.9779	0.0034	2.6715 (3.0861)	2.1703 (0.9123)	2.2885 (1.4237)	2.0182 (0.5487)	1.9735 (0.6200)	1.9672 (0.5710)	2.4342 (1.5301)	1.9927 (0.6607)	1.9493 (0.4489)	1.8784 (0.2874)	1.8651 (0.2489)	1.8159 (0.1681)
*q*_*H*_	0.1751	0.1699	0.2435 (0.2401)	0.2065 (0.0631)	0.2122 (0.1110)	0.1981 (0.0404)	0.1925 (0.0461)	0.1915 (0.0379)	0.2251 (0.1100)	0.1987 (0.0416)	0.1876 (0.0384)	0.1879 (0.0244)	0.1927 (0.0230)	0.1864 (0.0154)
*K*_*H*_2__	14.7208	15.0220	17.7895 (4.6844)	15.8602 (3.6790)	17.1807 (3.9218)	15.6285 (3.3153)	17.5520 (3.9027)	15.3650 (2.8692)	18.2597 (4.5231)	15.9178 (3.1224)	18.6338 (3.6628)	16.1130 (2.4214)	19.7661 (2.6491)	16.1545 (1.8235)
*P*_*D*_	3.1031	2.7916	7.3984 (8.1976)	4.1125 (2.3937)	4.1717 (3.2476)	3.6407 (1.7065)	4.5149 (4.3739)	3.4942 (1.1570)	4.3387 (2.7992)	3.2320 (1.1822)	4.3321 (4.9189)	3.2029 (0.9710)	3.5734 (1.2141)	3.1049 (0.7075)
*q*_*D*_	0.1639	0.1538	0.4269 (0.4866)	0.2346 (0.1461)	0.2336 (0.1899)	0.2054 (0.1022)	0.2424 (0.2365)	0.1995 (0.0708)	0.2373 (0.1616)	0.1876 (0.0706)	0.2360 (0.2723)	0.1840 (0.0597)	0.1998 (0.0746)	0.1780 (0.0432)
*K*_*D*_2__	14.1880	14.9758	15.5237 (8.7675)	15.2730 (6.4920)	17.7401 (8.0927)	15.3567 (5.8122)	15.7440 (5.9795)	13.9565 (3.6078)	16.7791 (6.1539)	15.3119 (3.1224)	16.4399 (6.0989)	14.9523 (4.0578)	14.9613 (3.9636)	14.6580 (2.9999)

*A significant locus is used to simulate 100 iterations with the NGE model at heritability levels of 0.05 and 0.1, and sample sizes of 66, 100, and 200, which have two genotypes: AA and Aa. The SE is the value in brackets*.

**Figure 8:**
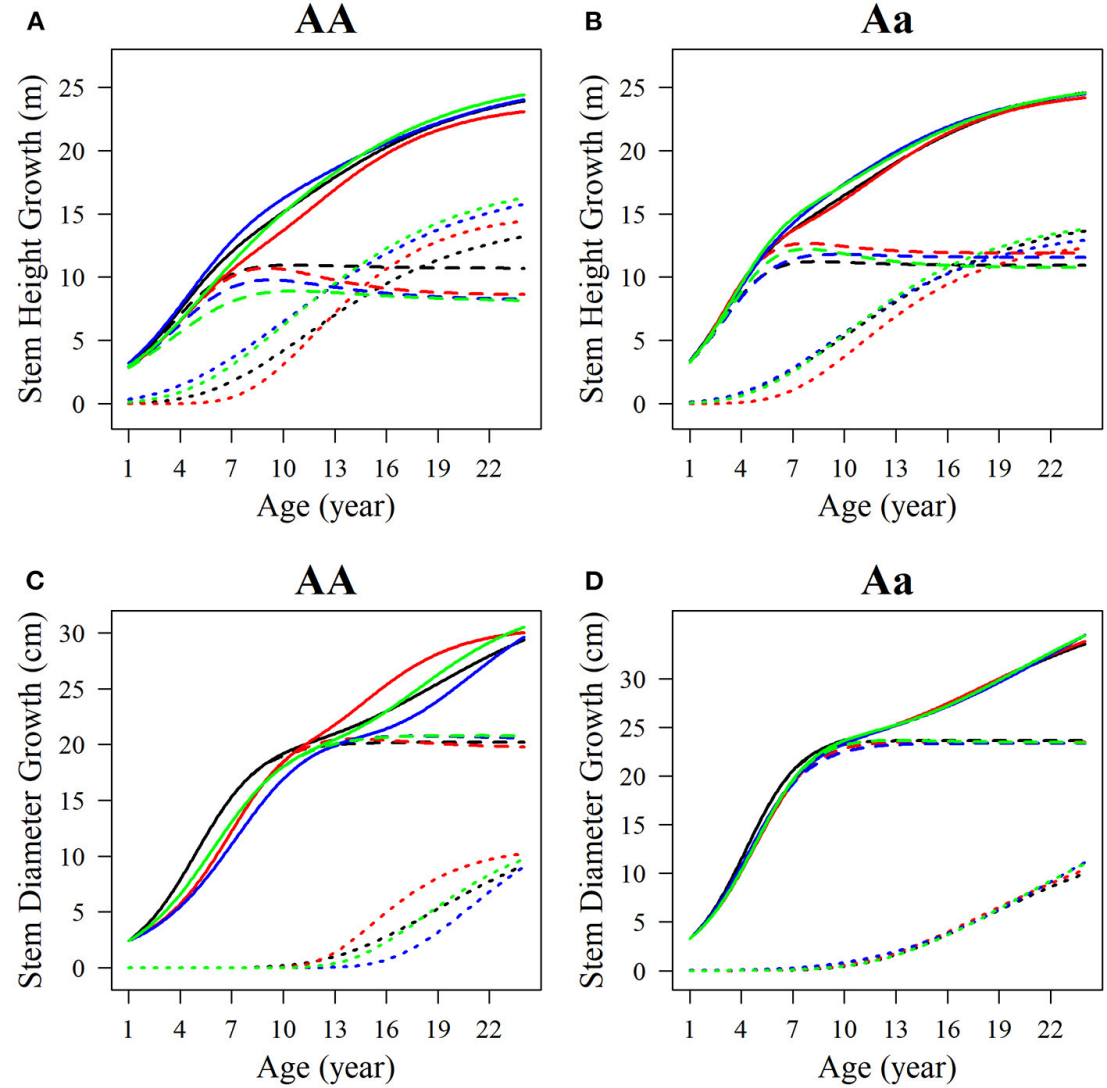
Growth curves of stem **(A, B)** height and **(C, D)** diameter of two genotypes with a heritability of 0.05. The overall growth (solid line) for each trait is decomposed into its first phase (broken line) and second phase growth components (dotted line). The sample sizes are 66, 100, and 200. Black represents the real curve, red represents the estimation curve with the sample size of 66, blue represents the estimation curve with the sample size of 100, and the green represents the estimation curve with the sample size of 200.

**Figure 9:**
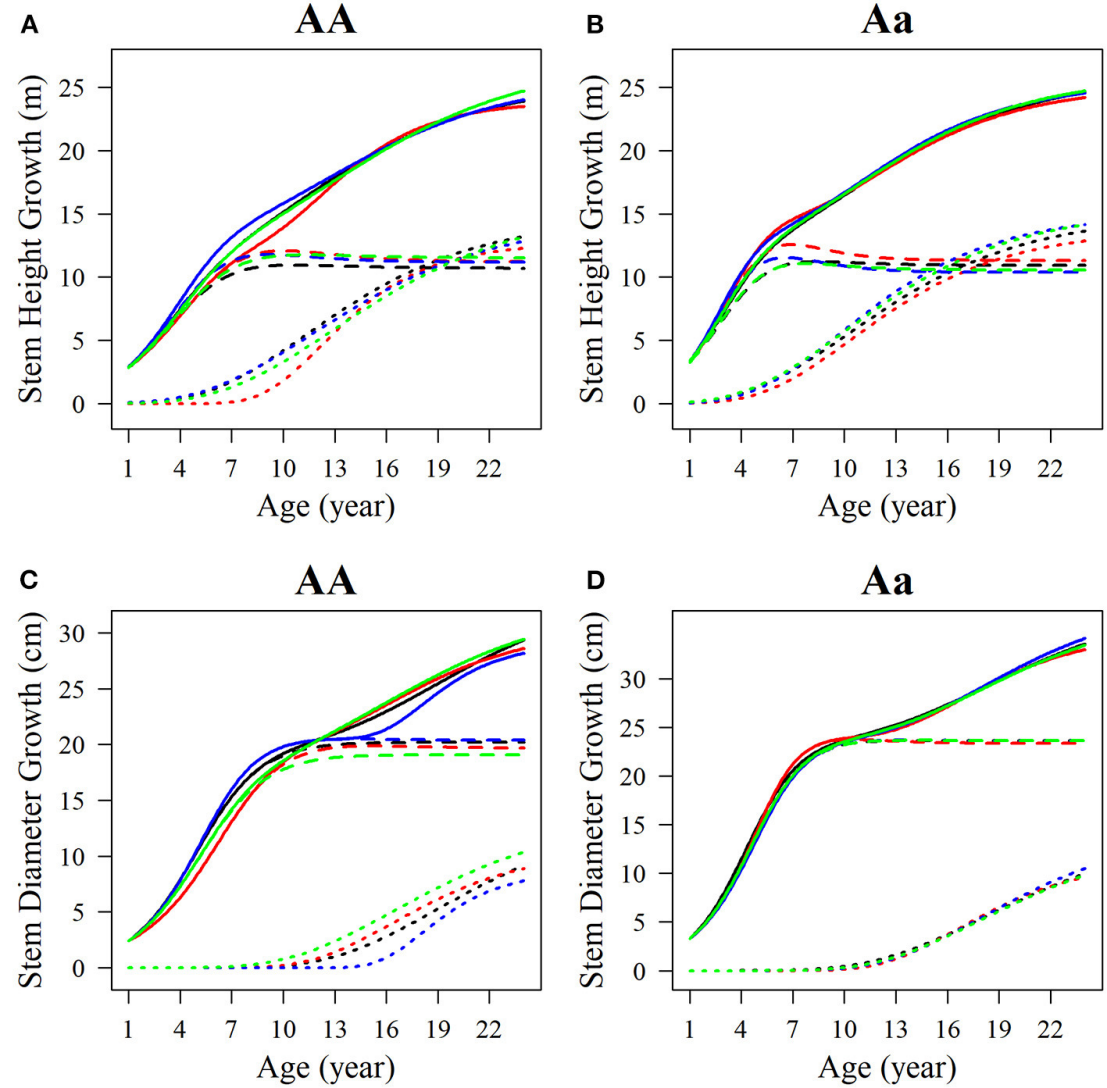
Growth curves of stem **(A, B)** height and **(C, D)** diameter of two genotypes with a heritability of 0.1. The overall growth (solid line) for each trait is decomposed into its first phase (broken line) and second phase growth components (dotted line). The sample sizes are 66, 100, and 200. Black represents the real curve, red represents the estimation curve with the sample size of 66, blue represents the estimation curve with the sample size of 100, and the green represents the estimation curve with the sample size of 200.

Among the estimated parameters of 100 groups of simulation data obtained under the six kinds of simulation conditions, we select several groups of parameters with better effects to produce the overall growth curves and growth curves of two phases of two interaction traits. The trends of the estimated curves of different simulation scales are consistent with those of the real curves (Figures 8, 9), which suggests that the genotype-specific curves estimated from the above example are reasonably convincing. By comparing the estimated curves of the two heritability levels, the simulation effect of the estimated curve of 0.1 heritability is obviously better than that of the estimated curve of 0.05 heritability. For heritabilities of both levels 0.05 and 0.1, the green estimated curve with 200 simulated quantities is the closest to the actual curve, and the simulation effect is the best, followed by the blue curve with 100 simulated quantities, and the red curve with 66 simulated quantities. As expected, the simulation effects increase with sample size. Larger sample sizes, such as 200, can effectively minimize noise and are suitable for QTL mapping using a multiphasic growth model.

## Discussion

The phase transition during tree growth is the internal mechanism for optimal development and reproduction. At the same time, it should not be ignored that phased development is not characterized by organ morphology and growth morphology independence, but there is a correlation between each morphology. For example, in the relationship between biomass yield and shrub growth traits, increasing the number of stems would reduce the average stem diameter but not the average stem length of plants (Mosseler et al., 2014). There are proportional relationships between stem cross-sectional area and stem weight, stem weight and leaf weight, and leaf weight and leaf area at the twig level (Sun et al., 2006). In our analysis of the phases of stem growth in trees, we emphasized the interaction between stem height and diameter during infancy, which has important biological significance. The NGE not only explored the continuous phase transitions that are difficult to distinguish in traditional analysis but also quantitatively described the interaction relationship between cooperation and confrontation among traits, thus further recognizing the individual functions of stem growth. We implemented systems mapping (Fu et al., 2017) to identify specific SNPs involved in multiphasic growth and provided a reliable ecophysiological perspective for understanding the growth of two sets of complex traits, tree height, and tree diameter.

The Gene Ontology (GO) analysis shows that the most significant SNPs can be annotated to genomic regions of candidate genes that encode particular biological processes (Supplementary Table S3). We find that a set of significant SNPs detected are highly linked within the same regions of chromosomes. Given that our mapping population is a full-sib family derived from two heterozygous parents, the linked SNPs, collectively acting as a QTL, may represent the same genes. A more precise characterization of the linked SNPs is needed through other genetic approaches.

The competitive or cooperative relationship between stem height and diameter is present throughout the growth history of trees, although it may take different forms during different phases and is sometimes not obvious. Our Lotka-Volterra-based interaction model can be extended to different phases of growth, describing complete tree growth and development and the interaction of traits within them. In addition, the overall multiphasic growth system of trees does not include only the interaction between the two traits. For example, the stem growth of trees also includes the growth of some lateral organs and branches in addition to the height and diameter of the stem. The compound phase growth model can be extended from a two-dimensional to a multidimensional model to conduct a comprehensive overall analysis of tree growth, although this expansion will greatly increase the complexity of the model and the difficulty of parameter estimation.

In the actual growth process, studies have shown that, in addition to endogenous regulatory factors, developmental genetic programs that control the transition between different phases of organisms are also regulated by environmental stimuli (Isabel and Dean, 2006; Huijser and Schmid, 2011; Srikanth and Schmid, 2011). Common environmental controls include stimuli such as temperature and photoperiod (Winfield et al., 2009; Kubota et al., 2014). For example, transcriptome changes in the transition of wheat from the vegetative to the reproductive growth stage are induced by cold and light (Winfield et al., 2009). Environmental factors affect the utilization of resources and morphological adaptation of trees to the environment, thus changing the competitive relationship between stem height and diameter and the growth and development phases. Therefore, our multiphasic framework can be extended to developmental transitions in different environments in order to study how stem growth structures respond to environmental changes. This theory can be used to analyze the interaction between QTLs and the environment with respect to the transformation of stem phase growth, and to analyze stem growth structure from the evolutionary point of view of tree adaptation to the ecological environment.

We performed many simulation analyses based on the actual data, and the QTL mapping method based on the composite multiphasic growth model exhibited good statistical characteristics and reasonably high specificity. Our composite multiphasic growth model provides an effective tool to describe the growth and development of tree diameter and height. In addition, relationship between phase development and internal growth is not limited to the stem growth structure of trees but also applies to the growth and phase transformation of quantitative traits associated with other organisms, such as root length, root thickness, and root number. The composite multiphasic growth model provides a reliable framework to map phylogenetic QTLs for phase change.

## Data Availability Statement

The original contributions presented in the study are included in the article/Supplementary Material, further inquiries can be directed to the corresponding author.

## Author Contributions

X-YZ conceived the idea and designed the model. HG performed data analysis and computer simulation and wrote the manuscript. SZ collected the data and annotated the gene. LJ designed the experiment and participated in the design of the data analysis. XZ tested the model. QF participated in data analysis and simulation. RW designed the data analysis. All the authors read and approved the manuscript.

## Funding

This study is supported by the Fundamental Research Funds for the Central Universities (No. BLX201912) (XZ) and the National Natural Science Foundation of China (grant no: 11501032) (X-YZ). This study is partially supported by the Japan Society for the Promotion of Science (grant no: 19K03613) (QF).

## Conflict of Interest

The authors declare that the research was conducted in the absence of any commercial or financial relationships that could be construed as a potential conflict of interest.

## Publisher's Note

All claims expressed in this article are solely those of the authors and do not necessarily represent those of their affiliated organizations, or those of the publisher, the editors and the reviewers. Any product that may be evaluated in this article, or claim that may be made by its manufacturer, is not guaranteed or endorsed by the publisher.
