# Supplementary material for: Genetic Architecture of Multiphasic Growth Covariation as Revealed by a Nonlinear Mixed Mapping Framework
Source: Front Plant Sci. 2021 Oct 5;12:711219. doi: 10.3389/fpls.2021.711219 (PMC8524055; doi:10.3389/fpls.2021.711219)
Supplement: Supplementary file 1 [file Data_Sheet_1.pdf]

Supplementary Material

1 Supplementary Figures and Tables

Table S1 Mean annual temperature and rainfall during 1987–2010 at Xuzhou, Jiangsu, China.

	1987	1988	1989	1990	1991	1992	1993	1994	1995	1996	1997	1998
Annual temperature (ºC)	14.7	14.6	14.5	14.8	14.1	14.4	14.2	15.5	14.7	14.4	15.1	15.4
Annual rainfall (mm)	51.71	41.72	57.40	99.07	65.16	74.05	69.70	68.43	68.78	80.48	66.19	94.08
	1999	2000	2001	2002	2003	2004	2005	2006	2007	2008	2009	2010
Annual temperature (ºC)	15.3	15.1	15.2	15.9	14.7	15.8	15.0	15.8	15.7	15.0	15.2	15.2
Annual rainfall (mm)	53.51	81.80	63.63	46.48	98.28	70.02	97.16	71.65	76.76	72.30	62.58	51.00

Table S2 Evaluation information for 1-24 year mean growth data fitted by diphasic growth models, including the NGE model, equation (2)-Logistic model and equation (2)-Richard model.

	NGE	equation (2)-Logistic	equation (2)-Richard
AIC	5.3991	5.4081	6.4453
BIC	5.7972	5.8062	6.9098
HQ	5.5564	5.5654	6.6288
R^2	0.9985	0.9985	0.9961
$adj.R^2$	0.9982	0.9982	0.9950
RSD	3.0006	3.0140	4.9115

Table S3 Physical locations, alleles, test P-values and numbers in the network of significant single nucleotide polymorphisms (SNPs) detected by the system mapping framework based on NGE.

Segregating types	Number	SNP	Line no.	Chr	Physical position	Allele	P-value	Type	Protein_id	Annotation
Intercross	1	17787	207608	1	46782762	A/G	4.83×10^{-62}	Noncoding		
Testcross	2	28821	326540	3	287037	G/A	3.75×10^{-48}	Noncoding		
Intercross	3	48502	546708	5	4510381	T/C	1.23×10^{-56}	exon	18207148	AT5G25830.1
Intercross	4	48561	546964	5	4549631	G/T	6.90×10^{-70}	Noncoding		
Testcross	5	48726	548063	5	4707454	T/C	4.32×10^{-46}	exon	18207567	AT5G63800.1
Intercross	6	48729	548070	5	4708309	A/G	1.90×10^{-58}	intron	18207567	AT5G63800.1
Intercross	7	48794	548688	5	4804826	G/C	6.43×10^{-61}	exon	18208487	AT3G52950.1
Intercross	8	48797	548706	5	4806540	C/A	1.27×10^{-64}	exon	18208487	AT3G52950.1
Intercross	9	48799	548712	5	4806812	A/G	1.27×10^{-64}	exon	18208488	AT3G52950.1
Intercross	10	48802	548722	5	4807469	C/T	2.05×10^{-60}	intron	18208487	AT3G52950.1
Intercross	11	48806	548729	5	4807703	A/T	2.50×10^{-57}	intron	18208487	AT3G52950.1
Intercross	12	48811	548739	5	4808398	A/G	1.15×10^{-70}	intron	18208487	AT3G52950.1
Intercross	13	48814	548745	5	4808912	G/T	6.88×10^{-60}	intron	18208487	AT3G52950.1
Intercross	14	48824	548760	5	4810033	A/C	7.78×10^{-62}	intron	18208487	AT3G52950.1
Intercross	15	48826	548762	5	4810091	A/G	7.62×10^{-62}	exon	18208487	AT3G52950.1
Intercross	16	48827	548763	5	4810136	T/A	1.27×10^{-64}	exon	18208487	AT3G52950.1
Intercross	17	48832	548775	5	4810932	T/G	2.50×10^{-62}	intron	18208487	AT3G52950.1
Intercross	18	48833	548776	5	4811009	A/G	2.29×10^{-60}	intron	18208487	AT3G52950.1
Intercross	19	48892	549457	5	4966044	A/C	1.69×10^{-61}	Noncoding		
Intercross	20	48916	549806	5	5053148	G/T	4.50×10^{-57}	Noncoding		
Intercross	21	48922	549823	5	5054573	T/A	1.85×10^{-58}	intron	18207561	AT2G22420.1
Intercross	22	48971	550358	5	5137853	C/A	2.12×10^{-71}	exon	18207812	AT4G39952.1
Intercross	23	73524	832200	8	790165	C/A	9.86×10^{-59}	intron	18249518	AT4G32605.1
Testcross	24	77477	868594	8	6595705	G/C	6.60×10^{-49}	exon	18247932	AT1G67620.1
Intercross	25	79620	889160	8	10214592	G/A	8.48×10^{-61}	intron	18248551	AT4G14880.1

Intercross	26	79624	889169	8	10215087	C/A	1.27×10^{-59}	intron	18248551	AT4G14880.1
Intercross	27	79625	889170	8	10215208	A/G	3.29×10^{-60}	intron	18248551	AT4G14880.1
Intercross	28	79662	889474	8	10281874	A/G	2.81×10^{-56}	exon	18249225	AT1G05460.1
Intercross	29	79688	889671	8	10306122	C/T	7.05×10^{-65}	exon	18249225	AT1G05460.1
Intercross	30	79745	890076	8	10382092	A/G	5.64×10^{-66}	Noncoding		
Intercross	31	79747	890087	8	10386508	T/C	3.22×10^{-58}	Noncoding		
Intercross	32	79748	890088	8	10386704	T/C	3.48×10^{-64}	Noncoding		
Intercross	33	79750	890130	8	10391300	A/G	1.52×10^{-64}	intron	18249295	AT4G34670.1
Intercross	34	79799	890555	8	10469303	A/G	3.43×10^{-60}	Noncoding		
Intercross	35	79812	890639	8	10493961	T/C	2.45×10^{-57}	Noncoding		
Intercross	36	79814	890653	8	10495448	A/C	6.61×10^{-67}	exon	18247875	AT3G22830.1
Intercross	37	79819	890785	8	10526702	C/T	4.70×10^{-59}	intron	18247554	AT1G60680.1
Intercross	38	79863	891157	8	10594229	T/C	3.85×10^{-60}	exon	18248726	AT4G00231.1
Testcross	39	85933	964239	9	8195744	A/G	8.06×10^{-47}	intron	18227200	AT3G19650.1
Testcross	40	85960	964624	9	8271390	C/T	1.58×10^{-48}	intron	18227905	NPH3
Testcross	41	85962	964627	9	8271597	C/A	8.06×10^{-47}	intron	18227905	NPH3
Testcross	42	85963	964633	9	8272417	C/G	5.85×10^{-53}	intron	18227905	NPH3
Testcross	43	86003	965071	9	8342740	A/G	8.06×10^{-47}	exon	18228881	AT1G49870.1
Testcross	44	86036	965297	9	8371779	T/G	8.06×10^{-47}	Noncoding		
Testcross	45	86037	965307	9	8374136	G/C	2.16×10^{-55}	intron	18228256	AT4G33925.1
Testcross	46	86043	965445	9	8399611	T/C	8.06×10^{-47}	Noncoding		
Testcross	47	86065	965668	9	8448217	T/G	2.85×10^{-48}	intron	18227796	AT2G14960.1
Testcross	48	86066	965669	9	8448252	T/A	3.18×10^{-46}	intron	18227796	AT2G14960.1
Testcross	49	86139	966225	9	8545931	G/T	7.04×10^{-47}	Noncoding		
Testcross	50	86348	968614	9	8959147	G/A	1.82×10^{-47}	exon	18227134	AT2G15620.1
Testcross	51	86354	968746	9	8990291	T/C	6.44×10^{-54}	exon	18228131	AT3G19260.1
Testcross	52	86976	974736	9	10010132	G/A	3.44×10^{-53}	exon	18228169	AT2G21520.2
Testcross	53	87023	975137	9	10076008	A/T	2.35×10^{-60}	Noncoding		

Testcross	54	87492	979087	9	10689746	G/T	7.75×10^{-53}	exon	18228564	AT4G34940.1
Testcross	55	87907	983081	9	11336448	T/G	7.30×10^{-58}	exon	18228525	AT4G35240.1
Intercross	56	102765	1160100	11	14632128	G/T	2.44×10^{-61}	exon	18230790	AT3G14620.1
Intercross	57	103635	1169276	11	16496034	T/C	2.58×10^{-59}	intron	18231226	AT4G27080.1
Intercross	58	110720	1244495	12	14207793	G/C	1.91×10^{-60}	intron	18228971	AT4G27080.1
Testcross	59	111175	1249435	13	248005	C/T	2.91×10^{-48}	Noncoding		
Testcross	60	111190	1249601	13	269980	A/C	3.80×10^{-54}	intron	18220676	AT3G62240.1
Testcross	61	117376	1319358	14	743904	A/G	1.83×10^{-46}	exon	18224157	GRF1
Testcross	62	117854	1323941	14	1662924	T/C	8.34×10^{-49}	intron	18222631	QWRF2
Testcross	63	137076	1548830	17	2837383	G/A	7.40×10^{-64}	Noncoding		
Testcross	64	154140	1783107	81	13104	C/T	5.91×10^{-50}	exon	18209048	AT3G48770.1
Intercross	65	154950	1812900	235	13915	T/C	1.76×10^{-57}	exon	18239519	AT3G51420.1

Table S4 Genotype NGE parameters of 65 significant single nucleotide polymorphisms (SNPs) detected by the system mapping framework. For the genotype of each significant SNP, the two rows of parameters in the table represent $\alpha_H, K_{H_1}, \beta_{H\leftarrow D}, \alpha_D, K_{D_1}, \beta_{D\leftarrow H}, p_H, q_H, K_{H_2}, p_D, q_D, K_{D_2}$ (separate Excel file).

2 Supplementary Figures

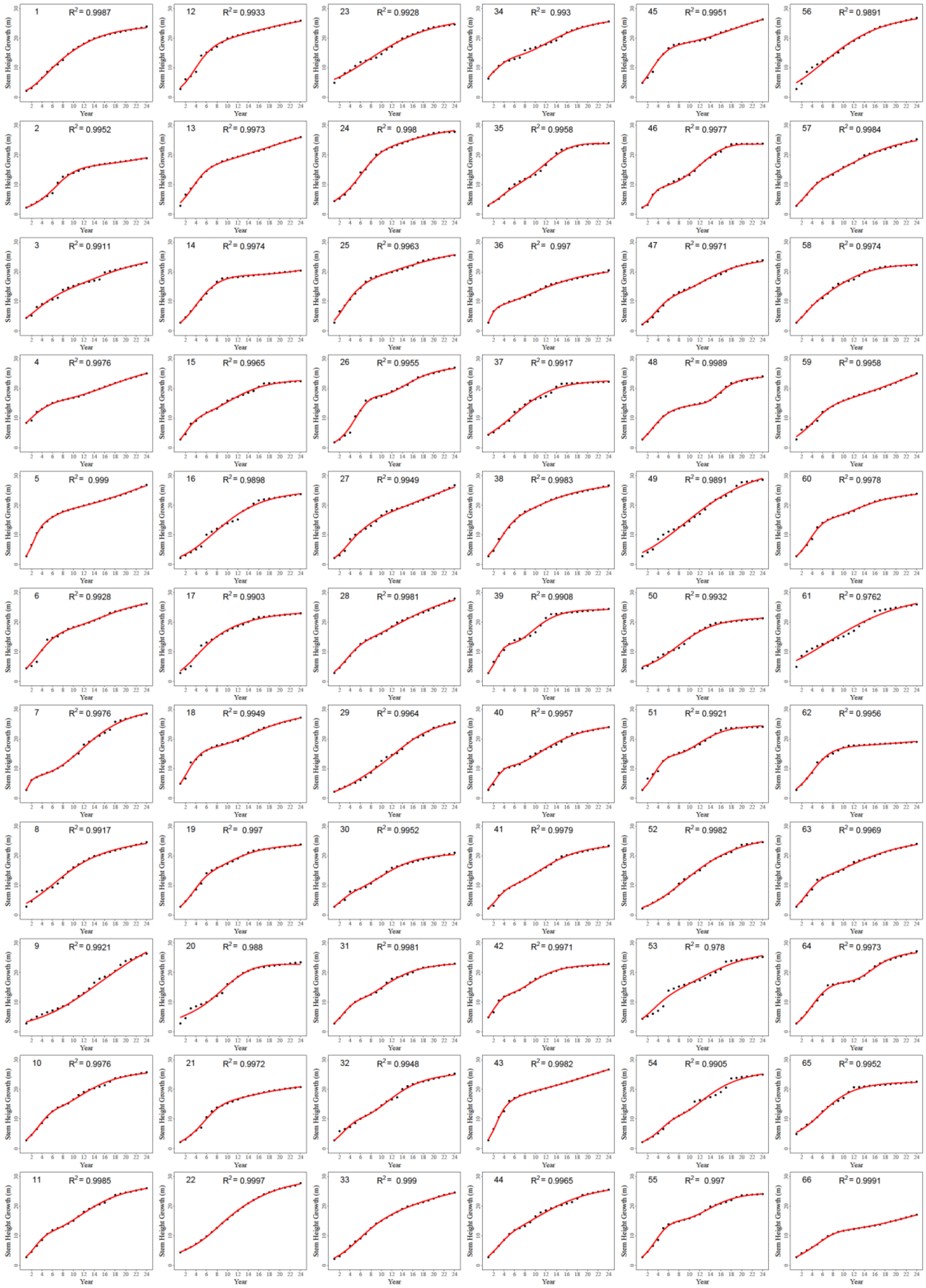

Figure S1 Fitted stem height growth curves (red line) of all progenies based on NGE, with raw data shown by black dots. R^2 is given for each tree.

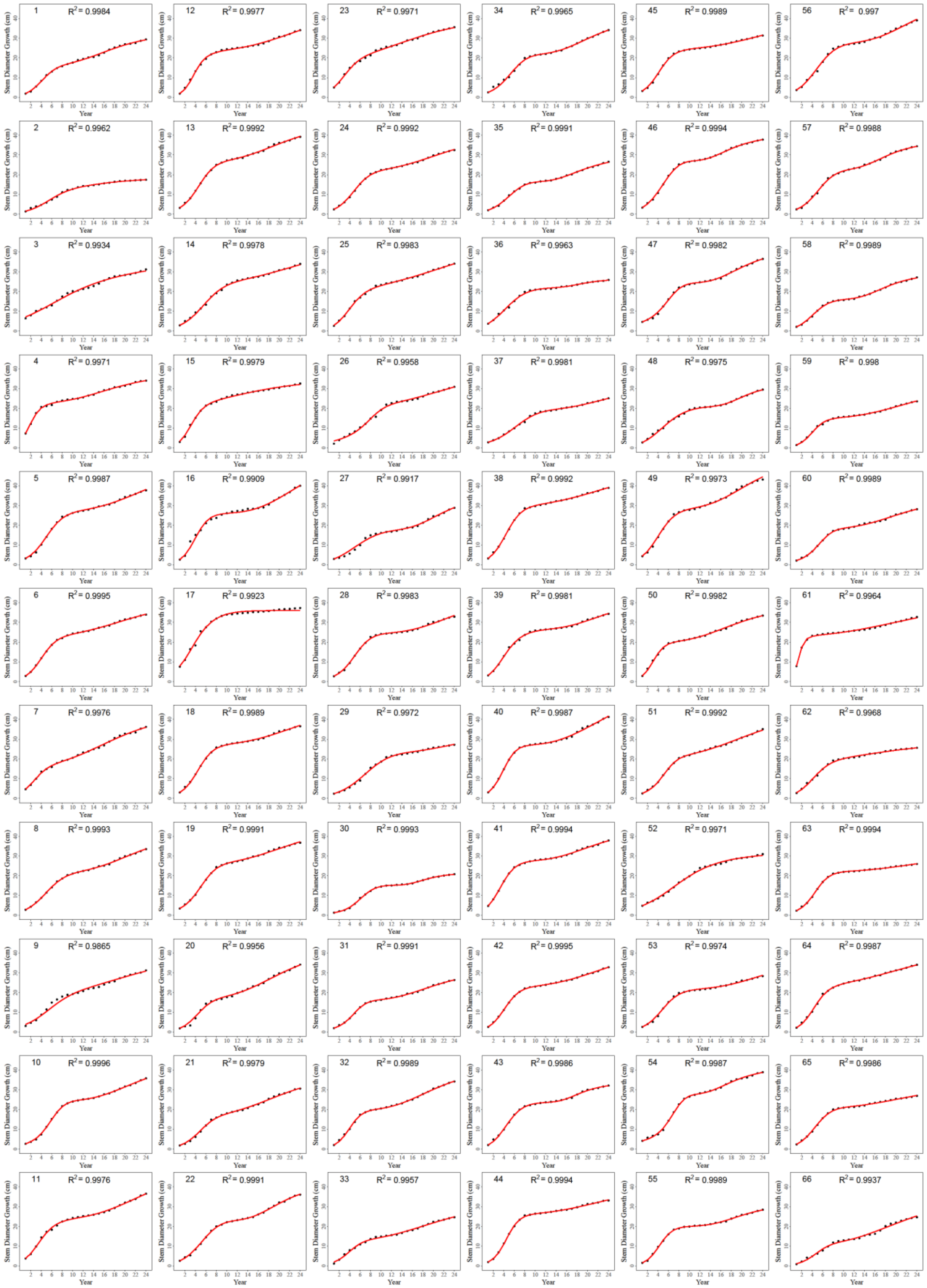
1 R2 = 0.9984
2 R2 = 0.9962
3 R2 = 0.9934
4 R2 = 0.9971
5 R2 = 0.9987
6 R2 = 0.9995
7 R2 = 0.9976
8 R2 = 0.9993
9 R2 = 0.9865
10 R2 = 0.9996
11 R2 = 0.9976
12 R2 = 0.9977
13 R2 = 0.9992
14 R2 = 0.9978
15 R2 = 0.9979
16 R2 = 0.9909
17 R2 = 0.9923
18 R2 = 0.9989
19 R2 = 0.9991
20 R2 = 0.9956
21 R2 = 0.9979
22 R2 = 0.9991
23 R2 = 0.9971
24 R2 = 0.9992
25 R2 = 0.9983
26 R2 = 0.9958
27 R2 = 0.9917
28 R2 = 0.9983
29 R2 = 0.9972
30 R2 = 0.9993
31 R2 = 0.9991
32 R2 = 0.9989
33 R2 = 0.9957
34 R2 = 0.9965
35 R2 = 0.9991
36 R2 = 0.9963
37 R2 = 0.9981
38 R2 = 0.9992
39 R2 = 0.9981
40 R2 = 0.9987
41 R2 = 0.9994
42 R2 = 0.9995
43 R2 = 0.9986
44 R2 = 0.9994
45 R2 = 0.9989
46 R2 = 0.9994
47 R2 = 0.9982
48 R2 = 0.9975
49 R2 = 0.9973
50 R2 = 0.9982
51 R2 = 0.9992
52 R2 = 0.9971
53 R2 = 0.9974
54 R2 = 0.9987
55 R2 = 0.9989
56 R2 = 0.997
57 R2 = 0.9988
58 R2 = 0.9989
59 R2 = 0.998
60 R2 = 0.9989
61 R2 = 0.9964
62 R2 = 0.9968
63 R2 = 0.9994
64 R2 = 0.9987
65 R2 = 0.9986
66 R2 = 0.9937
Stem Diameter Growth (cm)
Year

Figure S2 Fitted stem diameter growth curves (red line) of all progenies based on NGE, with raw data shown by black dots. R^2 is given for each tree.

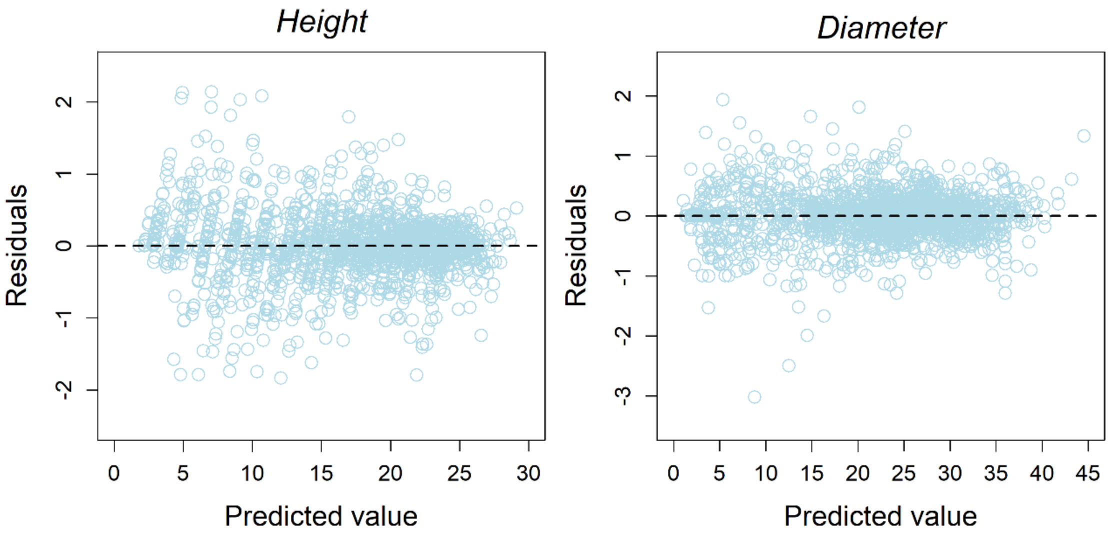

Figure S3 Random scatters of residuals over predicted values across each tree (circle) by NGE, warranting the statistical behavior of data fitting.

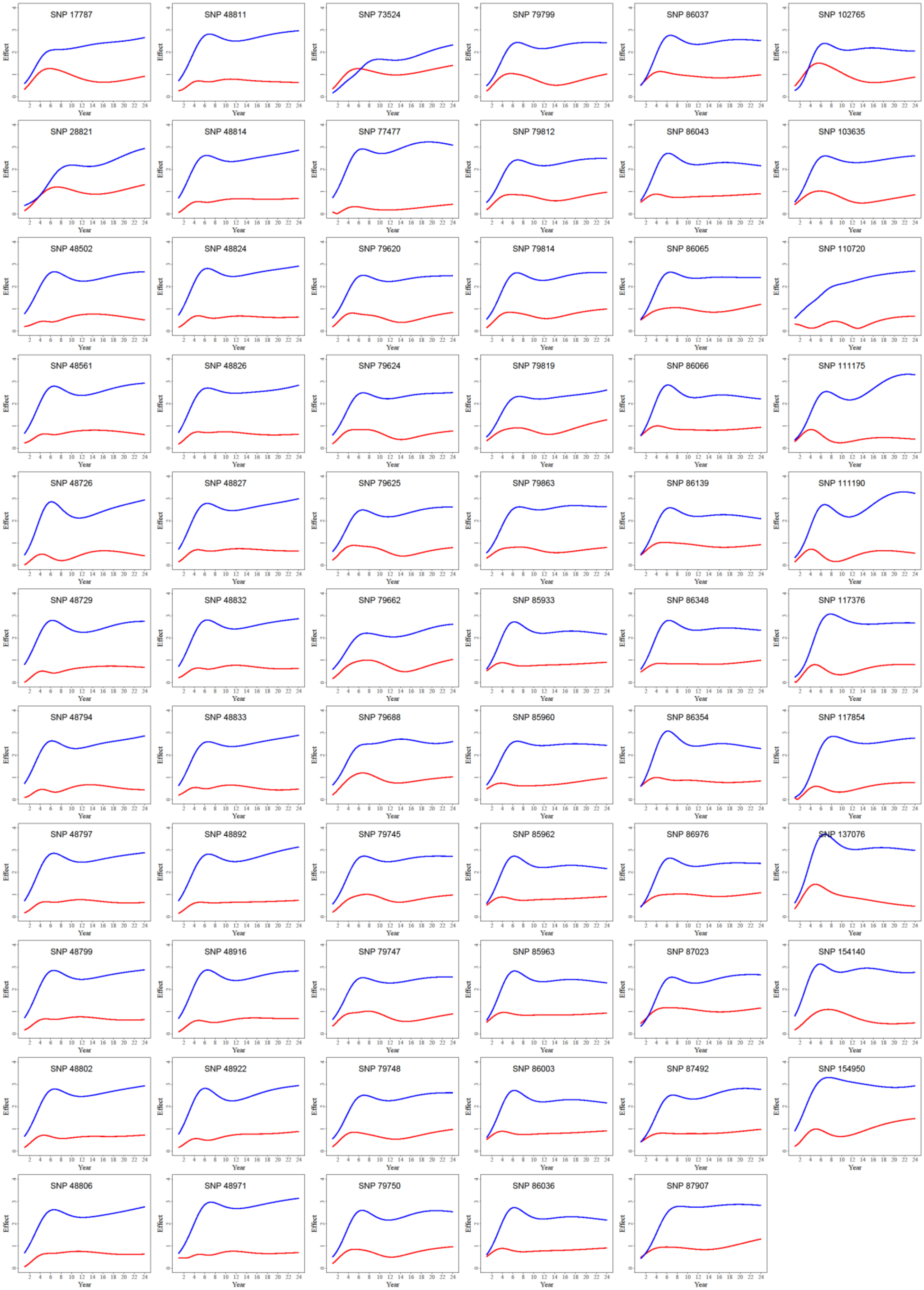
SNP 17787
SNP 48811
SNP 73524
SNP 79799
SNP 86037
SNP 102765
SNP 28821
SNP 48814
SNP 77477
SNP 79812
SNP 86043
SNP 103635
SNP 48502
SNP 48824
SNP 79620
SNP 79814
SNP 86065
SNP 110720
SNP 48561
SNP 48826
SNP 79624
SNP 79819
SNP 86066
SNP 111175
SNP 48726
SNP 48827
SNP 79625
SNP 79863
SNP 86139
SNP 111190
SNP 48729
SNP 48832
SNP 79662
SNP 85933
SNP 86348
SNP 117376
SNP 48794
SNP 48833
SNP 79688
SNP 85960
SNP 86354
SNP 117854
SNP 48797
SNP 48892
SNP 79745
SNP 85962
SNP 86976
SNP 137076
SNP 48799
SNP 48916
SNP 79747
SNP 85963
SNP 87023
SNP 154140
SNP 48802
SNP 48922
SNP 79748
SNP 86003
SNP 87492
SNP 154950
SNP 48806
SNP 48971
SNP 79750
SNP 86036
SNP 87907
Effect
Year

Figure S4 Genetic effect curves of 65 significant SNPs. The red lines represent the genetic effects of stem height and the blue lines represent the genetic effects of stem diameter.

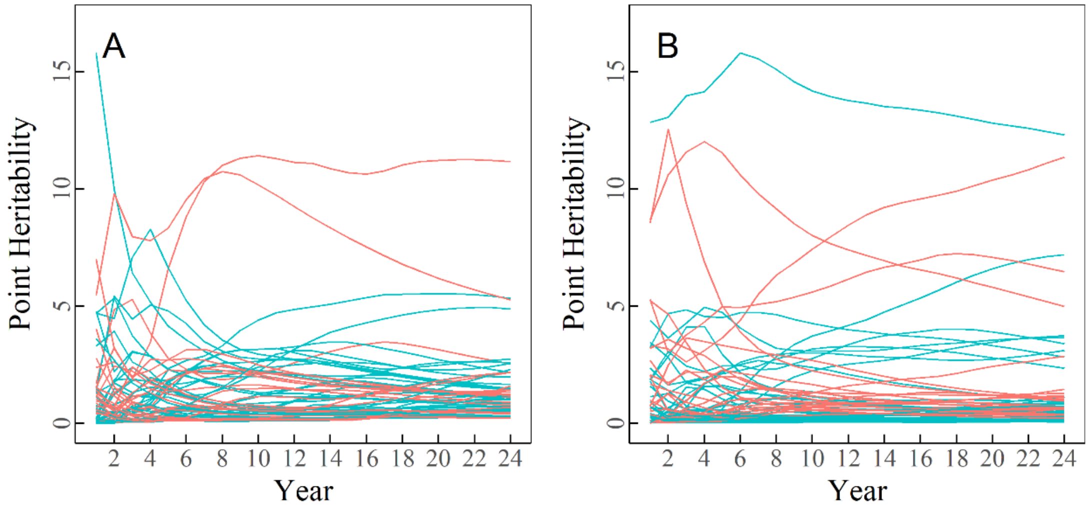

Figure S5. Heritability of stem height growth (A) and stem diameter growth (B) at different ages, explained by each of 65 significant SNPs. The cyan lines represent heritability of intercross markers, and red lines represent heritability of testcross markers.
